# Spatiotemporal assessment of health burden and economic losses attributable to short-term exposure to ground-level ozone during 2015–2018 in China

**DOI:** 10.1186/s12889-021-10751-7

**Published:** 2021-06-05

**Authors:** Zihan Zhang, Minghong Yao, Wenjing Wu, Xing Zhao, Juying Zhang

**Affiliations:** Department of Epidemiology and Biostatistics, West China School of Public Health and West China Fourth Hospital, Sichuan University, No.16 Section 3, Renmin South Road, Chengdu, 610044 China

**Keywords:** Ozone, Short-term, Health impact, Exposure factors, Economic loss, China

## Abstract

**Background:**

Ground-level ozone (O_3_) pollution is currently the one of the severe environmental problems in China. Although existing studies have quantified the O_3_-related health impact and economic loss, few have focused on the acute health effects of short-term exposure to O_3_ and have been limited to a single temporal and spatial dimension.

**Methods:**

Based on the O_3_ concentration obtained from ground monitoring networks in 334 Chinese cities in 2015–2018, this study used a two-stage exposure parameter weighted Log-linear exposure-response function to estimate the cause-specific mortality for short-term exposure to O_3_.

**Results:**

The value of statistical life (VSL) method that were used to calculate the economic loss at the city-level. Our results show that in China, the national all-cause mortality attributed to O_3_ was 0.27(95% CI: 0.14–0.55) to 0.39 (95% CI: 0.20–0.67) million across 2015–2018. The estimated economic loss caused by O_3_ was 387.76 (95% CI: 195.99–904.50) to 594.08 (95% CI: 303.34–1140.65) billion CNY, accounting for 0.52 to 0.69% of total reported GDP. Overall, the O_3_ attributed health and economic burden has begun to decline in China since 2017. However, highly polluted areas still face severe burden, and undeveloped areas suffer from high GDP losses.

**Conclusions:**

There are substantial health impacts and economic losses related to short-term O_3_ exposure in China. The government should pay attention to the emerging ozone pollution, and continue to strengthen the intervention in traditional priority areas while solving the pollution problem in non-priority areas.

**Supplementary Information:**

The online version contains supplementary material available at 10.1186/s12889-021-10751-7.

## Background

Ground-level ozone, as a secondary pollutant, is harmful to human health and crop yields. It is one of the six criteria air pollutants regulated by the U.S. Environmental Protection Agency [1, 2]. Generally, this gaseous pollutant is formed by the photochemical reaction of the precursors volatile organic compounds (VOCs) and nitrogen oxides (NO_x_), driven by solar radiation and temperature [3]. There are two primary sources of precursor material, including anthropogenic (e.g. transportation, coal burning, residential) and natural sources (e.g. plant, lightning, biochemical reactions in soil) [4]. In addition to emission sources, meteorological factors, the VOCs/NO_x_ ratio, and solar radiation can affect the formation of ozone [5–7]. Therefore, the diversity of sources and the uncertainty of influencing factors increase the difficulty of controlling ozone pollution.

In the past decades, with the development of China’s economy and urbanization, air pollution has become a public health issue of social concern. PM_2.5_ control, rather than ozone control, has become a national priority. In 2013, Chinese state council issued Air Pollution Prevention and Control Action Plan, which aims to solve the problem of particulate air pollution within five years by adopting ten strict measures, prioritizing the establishment of ten key city clusters, and setting specific limits for PM_2.5_ concentration in the Beijing-Tianjin-Hebei region (BTH), the Yangtze River Delta (YRD) and the Pearl River Delta (PRD) [8]. After five years of efforts, the PM_2.5_ mean concentration in China, the BTH, the YRD, the PRD region decreased by 39.4, 34.3, 27.7 and 39.6% from 2013 to 2017 [9]. In contrast, there is a continued increase in ground-level ozone concentration [10]. From 2013 to 2017, the annual ozone concentration in China, the BTH, the YRD, the PRD region increased by 20.4, 29.9, 22.1 and 8.6% respectively, while gaseous pollutant such as sulfur dioxide, nitrogen dioxide and carbon monoxide decreased by 12, 11 and 30% respectively during the same period in China [11]. In fact, ozone replaced particulate matter as the primary pollutant in the BTH, the YRD and the PRD region in 2016 [12]. Compared with Japan, South Korea, Europe and the United States, the magnitude and frequency of high-ozone events in China are much more significant [13].

In recent years, a series of epidemiological studies reported that short-term exposure to ozone is strongly associated with increased mortality risk of all-cause [14, 15], cardiovascular disease [16, 17] and respiratory disease [18]. Meanwhile, health burden assessment has been introduced to estimate the premature mortality and economic losses associated with ozone exposure. For example, Lin et al. estimated the number of COPD-related premature deaths attributable to long-term exposure to ozone in China in 2014, which was 89,391 (95% CI: 32,225 – 141,649) [19]. Liu et al. reported that the COPD deaths caused by long-term ozone exposure in China ranged from 55,341 to 80,280 in 2015 [12]. Maji et al. found that the national ozone attributable all-cause mortality was 69.6 (95% CI: 16.2–115) to 74.2 (95% CI: 16.7–127) thousand in 2016 [20]. Liang et al. estimated that 120 (95% CI: 67–160) thousand premature deaths could be avoided if China’s ground-level ozone concentration was reduced to the 100 μg/m^3^ [21]. Most of the existing studies focused on the long-term effects of chronic ozone exposure, while ignored the health impacts caused by short-term exposure, perhaps due to the long-term exposure effects is far significant than the short-term [22–27]. However, in China, even small short-term effects may cause huge health and economic burden due to the large population and high-intensity ozone exposure and it is still unclear the extent of the impact across the country. If this is a considerable number, then when considering the overall health burden related to ozone in China, the portion due to short-term exposure should be included and the corresponding economic losses should not be ignored. In addition, although existing studies have analyzed the spatial hotspots of ozone-related health and economic burden, for example, Maji et al. found that Beijing, Shanghai and Chengdu were the three cities with the highest number of premature deaths [20], these studies are limited to a single space dimension, and few explored the spatiotemporal patterns of health and economic burden caused by short-term exposure to ozone.

In epidemiological studies, exposure-response functions are used to link pollutant concentrations to their health effects, which are calculated based on exposure-response coefficients [28]. Therefore, accurate exposure-response coefficients can improve the accuracy of health burden estimation. In China, the exposure-response coefficient may vary depending on the local population, social and economic characteristics. However, most of the existing nationwide studies have set a single exposure-response coefficient for each health endpoint, which may bias the result [6, 29–31]. In addition, monetizing mortality attributable to air pollutants most commonly used the value of statistical life (VSL), while the willingness to pay (WTP) is the preferred method to calculate [32]. However, the spatial resolution of VSL in existing research can only be limited to the provincial level [20, 21, 30, 33]. Due to the economic imbalance in Chinese cities, the economically-developed cities may have higher VSL and dense population, and the coarse spatial resolution may not be conducive to the embodiment of economic loss differentiation between cities. As far as we know, no study introduced city-level VSL to estimate the economic loss attributable to air pollutants.

Exposure parameters are used to describe the amount or rate of human exposure to external pollutants (e.g. inhalation rate) as well as the basic characteristics of the human body (e.g. inhalation rate) and environment (e.g. floor area), which are important factors for accurate exposure assessment.

Due to the variety of local human activities, environment and social characteristics, the regional differences of exposure parameters may exist. If the exposure parameters are ignored, the spatial diversity of exposure dose on the inter-regional scale may be covered up. Therefore, the inclusion of localized exposure parameters in the health burden assessment can remedy this weakness. The first manual of exposure parameters was published in the United States as early as 1989 [34], while China did not publish the first edition until 2013. Most of the existing studies adopted the parameters of the United States [35, 36] or did not consider the modification effect of the parameters on outcomes at all [37, 38],which may lead to unreliable conclusions. For example, Wang et al. had found that if the inhalation rates of the United States were used directly in China, the error would be 2.6 to 30.9%, which would increase the uncertainty of health risk assessment results [39]. Zou et al. estimated the health burden of long-term exposure to PM_2.5_ and found that if exposure parameters were not taken into account, the number of premature deaths in China would have been overestimated by 12,434 to 14,684 cases [40].

Therefore, in order to estimate the spatiotemporal trend of health and economic burden caused by ozone short-term exposure in China in recent years, and to compensate the roughness of exposure-response coefficients selection and economic valuation in previous studies, as well as the estimation bias caused by ignoring exposure parameters. This study first estimated the premature deaths associated with short-term ozone exposure in 334 cities of China in 2015–2018 based on the Log-linear exposure-response function with the localized exposure-response coefficients. Second, we introduced localized exposure parameters to recalibrate the estimated number of premature deaths. Finally, we used the city-level VSL to convert the health burden into economic loss, and calculated the impact on local GDP. Our objectives are as follows: (1) to estimate city-specific health burdens and corresponding economic loss attributable to short-term O_3_ exposure; (2) to reveal the spatial and temporal dynamics of health and economic burden associated with short-term exposure to O_3_; (3) to propose enlightening information for decision-makers to develop ozone prevention and control strategies.

## Methods

### Data

#### Socio-economic and air pollutants data

We selected 334 cities covering 31 provinces of mainland China (Hong Kong, Macao and Taiwan were excluded due to the unavailability of data). The city-specific socio-economic data was derived from the 2015–2018 National Statistical Yearbook in each Province or the National Economic and Social Development Bulletins of each city, including permanent residents, per capita GDP, price index.

Data on daily ambient maximum 8-h average (DMA8) O_3_ concentration from 1st January 2015 to 31st December 2018 in 334 cities were retrieved through 1597 monitoring stations from the China National Environmental Monitoring Center (http://113.108.142.147:20035/emcpublish/), which provides real-time hourly air pollution concentrations to the public. We used the quality control measures of state-controlled monitoring data reported in the previous literature [41, 42]. The city-specific daily DMA8 O_3_ concentrations were all calculated based on the measurements from the available monitoring stations in each city [31, 43, 44]. According to ‘technical regulation for ambient air quality assessment of China’ (HJ 633–2013), we used the 90th percentile of DMA8 as the evaluation index of annual average daily maximum 8-h O_3_ concentration (ADMA8) for each city.

#### Data on baseline cause-specific mortality

“All-cause mortality”, “cardiovascular disease mortality”, “respiratory disease mortality” were selected as the health outcomes of our study due to the high correlation between these endpoints and ozone exposure in previous studies [45–48].

The daily baseline incidence rate of health outcomes was calculated as follows: Firstly, we obtained the corresponding annual all-cause mortality rate of 334 cities in 2015–2018 from the National Statistical Yearbook in each province and the city-specific National Economic and Social Development Bulletins (Fig. S1). Next, annual disease-specific mortality in each city can be obtained by multiplying annual all-cause mortality by the proportion of mortality rate in cardiovascular and respiratory disease [49]. The proportions of cardiovascular and respiratory disease mortality in all-cause death were derived from China Health and Family Planning Statistics Yearbook 2015–2018.

After getting the annual baseline incidence rate of health outcomes, we used the monthly mortality ratio of each province from the National Population Census (http://www.stats.gov.cn/tjsj/pcsj/rkpc/6rp/indexch.htm) to calculated the monthly mortality rate, then evenly allocated the monthly mortality rates to the daily mortality rates.

### Exposure parameters matching

Exposure parameters data were extracted from Chinese Environmental Exposure-Related Human Activity Patterns Survey (CEERHAPS) [50], a large national survey that began in 2011, which included a total of 91,527 samples aged ≥25 years and 5490 samples aged<5 years from 1908 villages, 636 towns, 159 counties and 31 provinces of mainland China. In this study, the inhalation rate and time spent outdoors were included in the analysis. Since the respiratory rate was relatively stable and the minimum spatial resolution was provincial, we matched the provincial annual mean value with corresponding province-governed cities. Similarly, we matched the time spent outdoors of each province with corresponding province-governed cities, but since the outdoor time takes different values in each season, we matched seasonal values. The provincial-specific statistics of these parameters were shown as Table. S1.

### Short-term exposure

The health impact of air pollutants to the population are classified as chronic health impact of long-term exposure and acute health impact of short-term exposure [16, 51]. Chronic health impact of long-term exposure to air pollutants usually refer to the health hazards of exposure to air pollutants for more than half a year, and relevant studies are mainly based on cohort studies [51, 52]. Cohort studies abroad have carried out earlier studies on the relationship between long-term exposure to air pollutants and different health outcomes, and a large number of research results have been accumulated. Only a few studies in China have reported the relationship between long-term exposure to air pollutants and premature death of different diseases [53–56]. Acute health impact of short-term exposure usually refers to the health hazards of the same day or a few days ago. Ecological time series [57], cluster study [58] and case cross study [59] are often used to explore the short-term health impact. There are a large number of studies on the relationship between short-term exposure to air pollutants and different health outcomes in China or foreign country, mainly focusing on non-accidental injury, death risk of respiratory and circulatory diseases [60–62], emergency visit, outpatient and hospitalization of respiratory and circulatory diseases [63–65], adverse pregnancy outcomes and mental health hazards [66, 67].

### Health impact assessment

As mentioned above, the introduction of exposure parameters into the analysis is a key step in accurately estimating the individual pollutant exposure and thus a more realistic reflection of the disease burden attributed to short-term exposure to O_3_. Therefore, different from previous studies [12, 68], this study adopted a two-stage analysis process. Firstly, as in previous studies [69–71], a Log-linear exposure-response function was adopted to estimate unadjusted health impact, which was recommended by WHO for health impacts assessment in severe pollution areas [72]. Additionally, the calculated health impacts were recalibrated at the city-level with matched exposure parameters to obtain a more accurate result.

In the first stage, the daily health outcomes attributable to O_3_ in each city was evaluated as follows [20, 30, 73–75]:

1 $$ H{I}_{i,j, city}={BI}_{i,j, city}\ast {EP}_{city}\ast {ER}_{i,j, city} $$

Where *HI*_*i*, *j*, *city*_ represents the health outcome *j* attributed to short-term O_3_ exposure on day *i*, *B*_*i*, *j*, *city*_ is the baseline incidence rate of corresponding health outcome *j* on day *i*, *EP*_*city*_ is the target population, *ER*_*i*, *j*, *city*_ denotes the excess risk of health outcome *j* on day *i*.

The *ER*_*i*, *j*, *city*_ mentioned above was estimated by Log-linear exposure-response function, which has been widely used to assess the acute health effects of air pollution for environmental epidemiological studies in China [15, 76]:

2 $$ {ER}_{i,j, city}=\mathit{\exp}\left[{\beta}_{j, city}\left({C}_{i, city}-{C}_o\right)\right]-1 $$

Where *β*_*j*, *city*_ is the exposure-response coefficient of health outcome *j* obtained from the recent epidemiological studies in China (Table. 1). If the corresponding coefficient of a province cannot be found in published epidemiological studies, the data closest to that province would be selected as the substitute value. If there are no similar provinces, the results of national meta-analysis would be used as the substitute value. *C*_*i*, *city*_ is the city-specific daily ambient maximum 8-h average O_3_ concentration, and *C*_*o*_ is threshold concentration representing no adverse health effect when the exposure concentration is below *C*_*o*_ which was assumed to be zero for our study [77–79]. The uncertainty was estimated based on a Monte Carlo simulation. It produced 1000 sets of coefficients and ERFs from which 1000 sets of premature mortality estimations were calculated. Then, we used the 50, 2.5, and 97.5% values of the simulated results as the estimation of the central value, lower, and upper *CI* values, respectively.

**Table 1:** Exposure-response coefficients for the short-term health impacts of O_3_

Cause-specific mortality	RR (95%CI) (10 μg/m3)	Coefficient β	Study region	References
All-cause	1.0037 (1.0020–1.0055)	3.7E-04 (2.0E-04 – 5.5E-04)	34 Chinese cities	(Sun et al. 2018) [1]
	1.0042 (1.0032–1.0052)	4.2E-04 (3.2E-04 – 5.2E-04)	7 Chinese cities	(Yan et al. 2013) [2]
	1.0036 (1.0012–1.0060)	3.6E-04 (1.2E-04 - 6.0E-04)	East China	(Madaniyazi et al. 2016) [75]
	1.0055 (1.0034–1.0076)	5.5E-04 (3.4E-04 - 7.6E-04)	Jiangsu	(Chen et al. 2017) [15]
	1.0045 (1.0016–1.0730)	4.5E-04 (1.6E-04 - 7.3E-03)	Shanghai	(Zhang et al., 2006) [5]
	1.0038 (1.0023–1.0053)	3.8E-04 (2.3E-04 - 5.3E-04)	Wuhan	(Wong et al. 2008) [18]
	1.0056 (1.0042–1.0074)	5.6E-04 (4.2E-04 - 7.4E-04)	Xi’an	(Zhong et al. 2017) [7]
	1.0024 (1.0013–1.0035)	2.4E-04 (1.3E-04 - 3.5E-04)	Nationwide	(Yin et al. 2017) [8]
Cardiovascular	1.0039 (1.0016–1.0062)	3.9E-04 (1.6E-04 – 6.2E-04)	34 Chinese cities	(Sun et al. 2018) [1]
	1.0044 (1.0017–1.0070)	4.4E-04 (1.7E-04 – 7.0E-04)	7 Chinese cities	(Yan et al. 2013) [2]
	1.0060 (1.0022–1.0097)	3.8E-04 (2.3E-04 - 5.3E-06)	East China	(Madaniyazi et al. 2016) [75]
	1.0098 (1.0058–1.0137)	9.8E-04 (5.8E-04 - 1.4E-03)	Jiangsu	(Zhang and Zhang 2019) [9]
	1.0053 (1.0010–1.0096)	5.3E-04 (1.0E-04 - 9.6E-04)	Shanghai	(Zhang et al. 2006) [5]
	1.0037 (1.0001–1.0073)	3.7E-04 (1.0E-05 - 7.3E-04)	Wuhan	(Wong et al. 2008) [18]
	1.0027 (1.0010–1.0044)	2.7E-04 (1.0E-04 - 4.4E-04)	Nationwide	(Yin et al. 2017) [8]
Respiratory	1.0050 (1.0022–1.0077)	5.0E-04 (2.2E-04 – 7.7E-04)	7 Chinese cities	(Yan et al. 2013) [2]
	1.0051 (1.0003–1.0098)	5.1E-04 (3.0E-05 - 9.8E-04)	East China	(Madaniyazi et al. 2016) [75]
	1.0131 (1.0045–1.0217)	1.3E-03 (4.5E-04 - 2.2E-03)	Shanghai	(Chen et al. 2010) [10]
	1.0034 (1.0001–1.0075)	3.4E-04 (1.0E-05 - 7.5E-04)	Wuhan	(Wong et al. 2008) [18]
	1.0073 (1.0049–1.0097)	7.3E-04 (4.9E-04 - 9.7E-04)	Nationwide	(Shang et al. 2013) [16]

In the second stage, we use a weighted coefficient *W*_*i*, *city*_ composed of matched exposure parameters to calibrate the health impact of each city. According to Zou’s study [40], *W*_*i*_ was evaluated as Eq. (3):

3 $$ {W}_{i, city}=\frac{l_{city}}{l_{ave}}\ast \left(\frac{t_{i, city}+{\beta}_i\left(24-{t}_{i, city}\right)}{t_{ave}+{\beta}_i\left(24-{t}_{ave}\right)}\right) $$

For each city, *l*_*city*_ represents the average inhalation rates of the province to which the city belongs. Where *l*_*ave*_ is the national average value of the inhalation rates across mainland China. Correspondingly, *t*_*i*, *city*_ is equal to the average time spent outdoors on day *i* and *t*_*ave*_ means the overall average time spent outdoors in mainland China. It should be noted that since the minimum time resolution is at the seasonal level, there are only four values of *t*_*i*, *city*_, corresponding to the values of spring, summer, autumn and winter respectively. Finally, *β*_*i*_, the permeability coefficient, represents the ratio of indoor O_3_ concentration to outdoor O_3_ concentration on day *i*. Since there is no research on measuring I/O ratio of O_3_ in season in China, we use the results of Yang’s study to approximately replace the I/O ratio of each season. We use the I/O ratio measured when the window is opened as the data in summer, we use the I/O ratio measured when the window is closed as the data in winter, and we use the average of I/O ratio as the data in spring and autumn (because in China, the temperature in spring and autumn is close, and the temperature is between summer and winter) [80].

Eventually, the daily health impacts adjusted for exposure factors in each city are calculated by multiplying the weight coefficients by the unadjusted health impacts:

4 $$ {HI}_{i,j, city}^{\ast }=H{I}_{i,j, city}\ast {W}_{i, city} $$

By summing up the adjusted health impacts of each day in year k, the health impacts *j* of the city in year k attributable to short-term exposure to O_3_ can be estimated:

5 $$ {HI}_{k,j, city}=\sum \limits_{i=1}^{365}{HI}_{i,j, city}^{\ast } $$

### Estimation of economic loss

For pollutant-related mortality, the value of statistical life (VSL) method and the human capital approach (HCA) are commonly used to estimate the economic loss in previous studies. We selected VSL method because HCA does not take into account non-use values such as the individuals’ spiritual damage, which is considered as the lower bound of the economic loss estimation [72]. In China, there are few studies estimated the original VSL of a specific city. Therefore, we used the benefits transfer method to estimate the city-specific VSL from a developed city (e.g. Beijing) by adjusting per capita GDP and price index [81] (Fig. S2). City-specific VSL per person in year k can be constructed by Eq. (6):

6 $$ {VSL}_{k, city}={VSL}_b\ast {\left(\frac{G_{city,2010}}{G_{2010}}\right)}^{\beta }{\left(1+\%\varDelta {P}_{k, city}+\%\varDelta {\gamma}_{k, city}\right)}^{\beta } $$

Where VSL_*k*, *city*_ is the adjusted VSL for a specific city in year k, VSL_*b*_ is the value of statistical life for Beijing in 2010 estimated by Xie [82] and its value is 1.68 million Yuan, *β* is the income elasticity of health cost equals to 0.8 as recommended by OECD [83], %ΔP_*k*, *city*_ is the percentage increase of consumer price in specific city from year 2010 to year k. This is measured by consumer price index (CPI) that reflects the inflation. %Δγ_*k*, *city*_ means the percentage change in real GDP per capita growth in a specific city from 2010 to year k. This is derived from real GDP per capita annual growth. Finally, we can calculate that the city-specific per capita VSL and the economic loss due to outcome *j* in year k can be calculated by the following formula:

7 $$ {EL}_{k,j, city}={VSL}_{k, city}\ast {HI}_{k,j, city} $$

We need to emphasize that the uncertainty calculation in this study only considered the uncertainty of the model coefficient estimation. Due to the data limitation, this calculation did not include the uncertainty of ground-level O_3_ concentrations, population data, baseline mortality rates, VSL estimation, which may underestimate the uncertainty of the mortality/economic burden estimates.

## Results

### Ozone exposure assessment

**Fig. 1:**
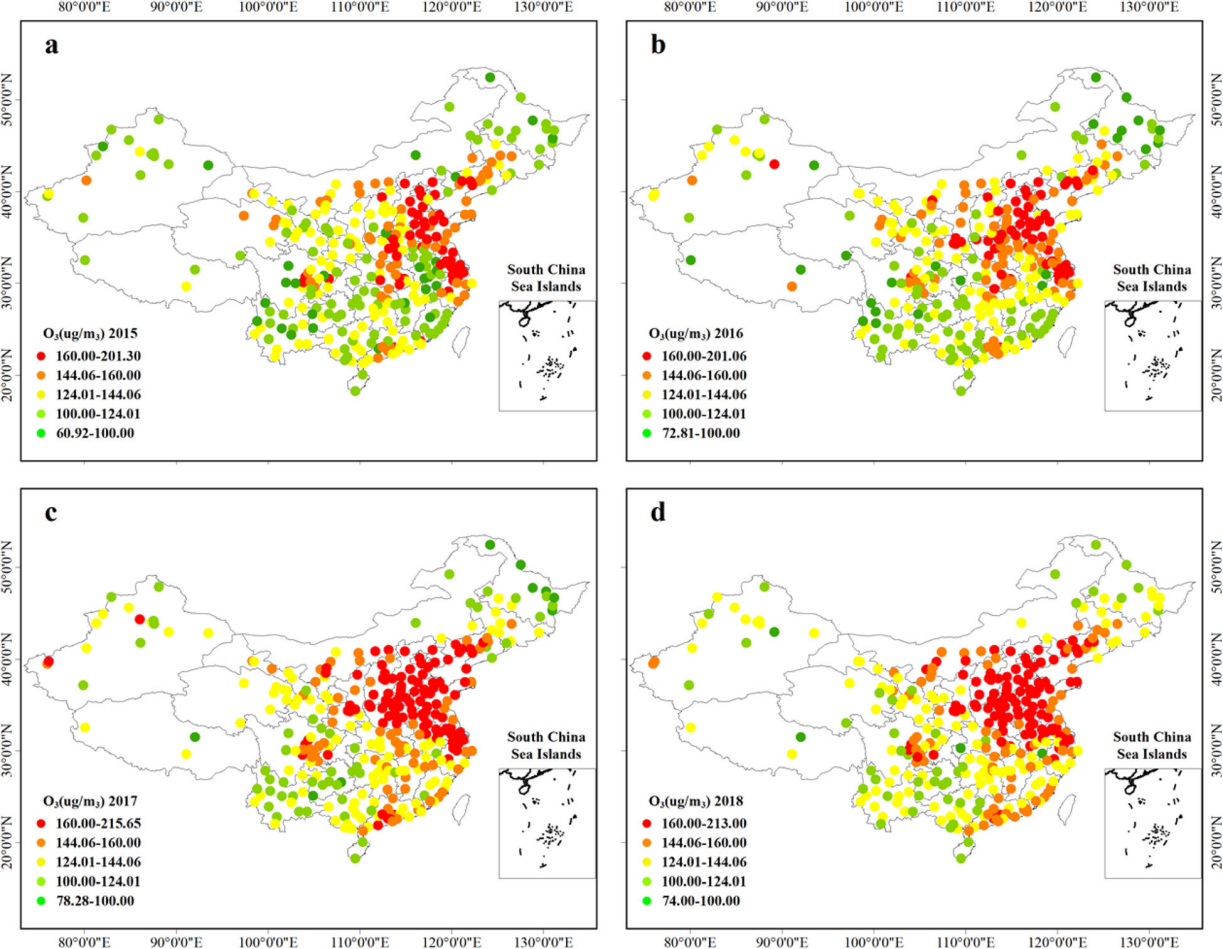
The city-specific annual 90th percentile daily maximum 8-h O3 concentrations in 2015 (**a**), 2016 (**b**), 2017 (**c**) and 2018 (**d**). The map was generated using the ArcMap 10.5 software, and the shape file were built-in resources of the software

**Fig. 2:**
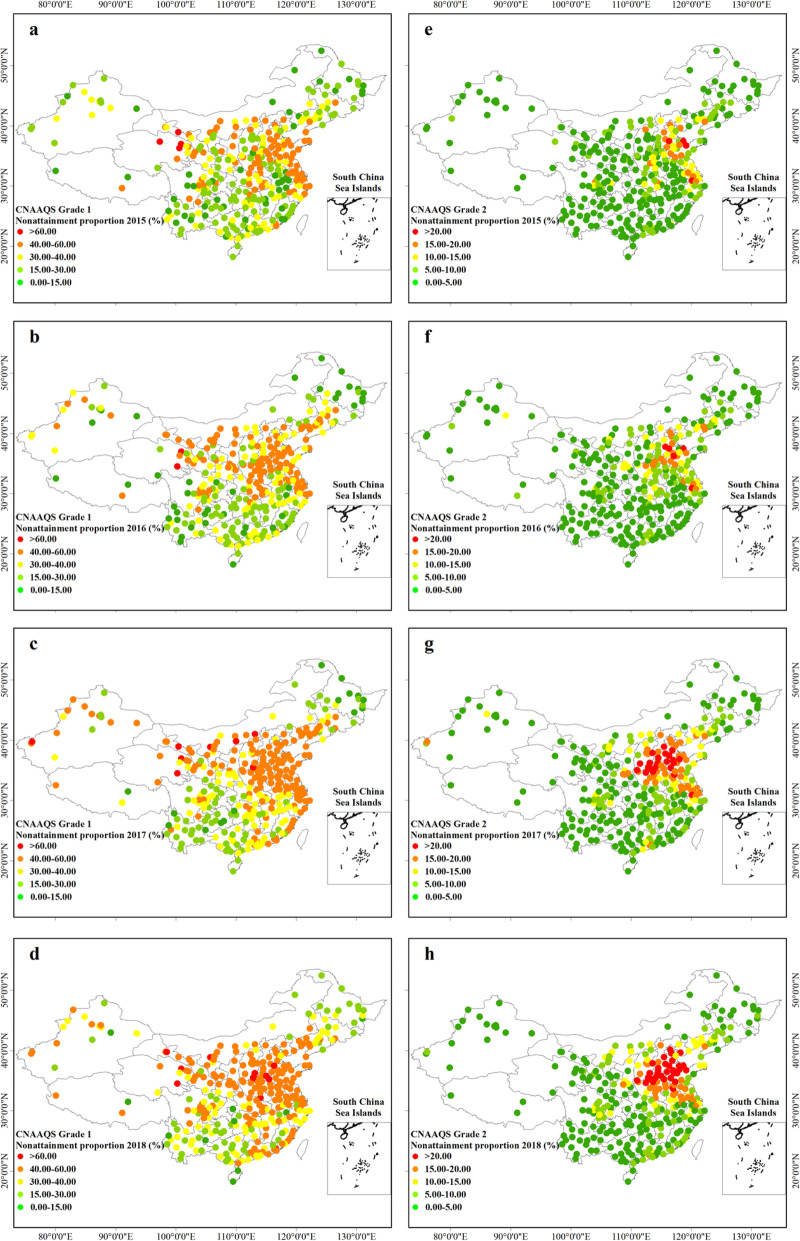
The city-specific nonattainment rate of CNAAQS Grade I (**a - d**) and Grade II (**e - h**) during 2015–2018. The map was generated using the ArcMap 10.5 software, and the shape file were built-in resources of the software

The annual daily maximum 8-h O_3_ concentrations (DMA8) in 334 cities between 2015 and 2018 are shown in Fig. 1. It can be seen that the ozone concentration in the north was higher than that in the south, and the Beijing-Tianjin-Hebei (BTH), the Yangtze River Delta (YRD), the Pearl River Delta (PRD), the Chengdu-Chongqing city belt and the Central Plains city cluster were the major polluted areas. In 2015, the national ozone level ranged from 60.92 μg/m^3^ (in Chuzhou in Anhui province) to 201.30 μg/m^3^ (in Beijing), with an average of 133.94 ± 26.13 μg/m^3^, which was 137.07 ± 24.96 μg/m^3^ (72.81 μg/m^3^ in Deqen in Yunnan to 201.06 μg/m^3^ in Huzhou in Zhejiang), 149.07 ± 27.31 μg/m^3^ (72.28 μg/m^3^ in Naqu in Tibet to 215.65 μg/m^3^ in Baoding in Hebei) and 149.16 ± 25.51 μg/m^3^ (74.00 μg/m^3^ in Turpan in Xinjiang to 213.00 μg/m^3^ in Liaocheng in Shandong) respectively in 2016, 2017 and 2018. The daily maximum 8-h O_3_ concentrations (DMA8) based on monitoring data during 2015–2018 were analysed and compared with the China National Ambient Air Quality Standards (CNAAQS). Cities in Qinghai and Gansu province had a high ratio of days exceeding the CNAAQS Grade I (100 μg/m^3^) (Fig. 2a), whereas the BTH, YRD regions and Shandong Peninsula had a high proportion of days violating the CNAAQS Grade II (160 μg/m^3^) (Fig. 2b). Population distribution and O_3_ concentration distribution had the same pattern that concentration was higher in the densely populated regions than those in sparsely populated regions (Fig. S3). 95.81 and 25.46% of the population lived in regions where annual O_3_ concentrations exceeded CNAAQS Grade I (100 μg/m^3^), Grade II (160 μg/m^3^) criteria in 2015. In 2016, 2017 and 2018, the figures were 96.41 and 26.22%, 99.17 and 45.83%, 99.78 and 45.47%. Besides, we found that from 2015 to 2016, the ozone concentration in BTH region, Central Plains City belt, Guan Zhong City belt, Yangtze-Huaihe City belt experienced a dramatically increase, while the concentrations in southwest, northeast and coastal areas of China decreased (Fig. S4a). In 2016–2017, ozone concentrations increased across the country (Fig. S4b). From 2017 to 2018, regions with a significant increase in ozone concentration in the previous year generally declined (Fig. S4c), which suggested that the government was implementing spatial–differentiation air pollution control strategies.

### Ozone-attributed premature mortality

**Fig. 3:**
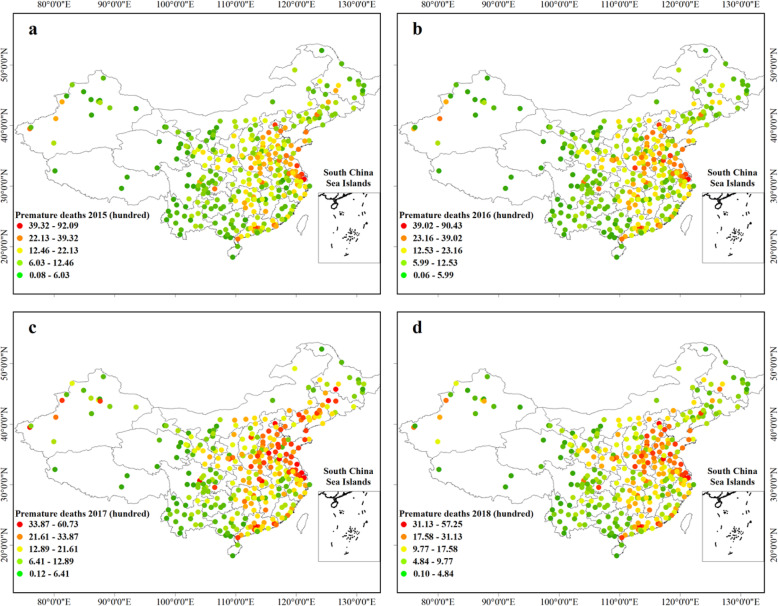
The city-specific O3-realted all-cause mortality in 334 Chinese cities in 2015 (**a**), 2016 (**b**), 2017 (**c**) and 2018 (**d**). The map was generated using the ArcMap 10.5 software, and the shape file were built-in resources of the software

All-cause premature deaths attributed to O_3_ in 334 cities in China are shown in Fig. 3. The total premature deaths attributable to O_3_ in 334 cities was 0.27 (95% CI: 0.14–0.55) million, 0.28 (95% CI: 0.15–0.56) million, 0.39 (95% CI: 0.20–0.67) million, 0.32 (95% CI: 0.16–0.57) million in 2015, 2016, 2017, 2018 respectively. There were 92 (27.5%), 106 (31.7%), 147 (44.0%), 126 (37.7%) cities with more than 1000 deaths in 2015, 2016, 2017, 2018 respectively, most of which are located in the northern and central parts of China. In 2015, the three cities with the highest all-cause mortality were Shanghai [9.21 (95% CI: 3.27–149.90) thousand], Guangzhou [3.93 (95% CI: 2.58–5.28) thousand] in Guangdong Province and Taizhou [3.72 (95% CI: 2.30–5.14) thousand] in Jiangsu Province. By 2018, Shanghai [5.73 (95% CI: 2.04–93.19) thousand], Guangzhou [4.99 (95% CI: 3.28–6.71) thousand] and Beijing [4.45 (95% CI: 1.48–7.41) thousand] were the three cities with the highest number of ozone-related deaths. Generally, these cities in densely populated areas such as the YRD and PRD will naturally face a higher health burden. However, we found that there was also severe a health burden in some sparsely populated cities, such as Ili and Kashgar in 2015, Aksu in 2016, Kashgar, Ili, Wujiaqu, Aksu, Bayannur in 2017, Ili, Kashgar, Wujiaqu in 2018. (> 1000 people per city).

**Fig. 4:**
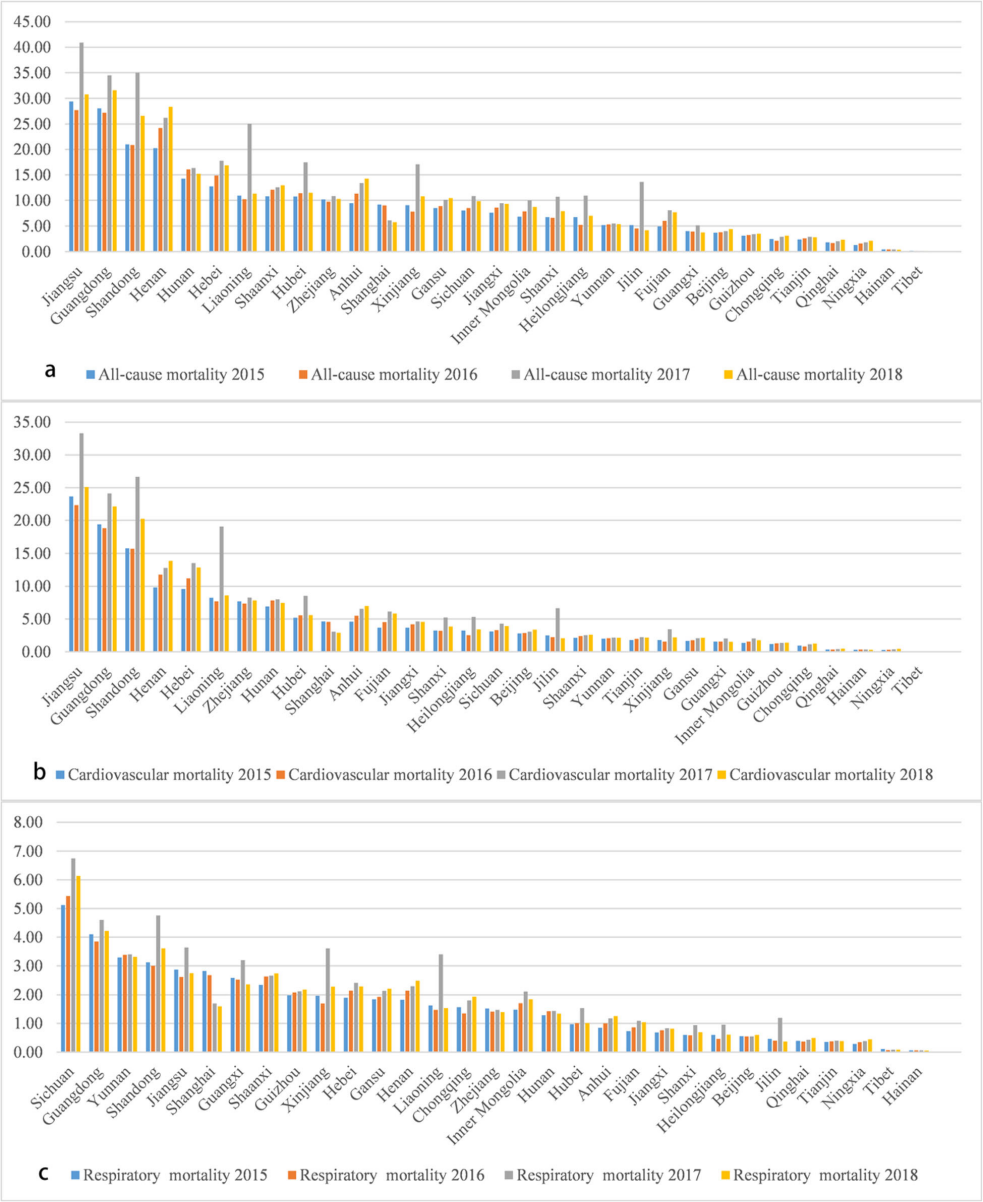
Provincial-level all-cause (**a**), cardiovascular (**b**) and respiratory (**c**) disease premature death (thousand) due to ozone short-term exposure during 2015–2018

Fig. S5, S6 illustrates the spatial distribution of premature mortality from cardiovascular and respiratory disease due to short-term ozone exposure in China in 2015–2018.The cardiovascular mortality related to O_3_ was 0.15 (95% CI: 0.05–0.27) million, 0.16 (95% CI: 0.05–0.27) million, 0.22 (95% CI: 0.07–0.38) million, 0.18 (95% CI: 0.06–0.31) million in 2015, 2016, 2017, 2018 in China, respectively. Respiratory disease mortality account for about one third of cardiovascular disease. In 2015, 2016, 2017 and 2018, the number was 49.91 (95% CI: 19.09–82.25) thousand, 50.35 (95% CI: 19.32–83.02) thousand, 63.18 (95% CI: 23.16–105.36) thousand and 54.11 (95% CI: 20.88–89.16) thousand respectively. Different from the all-cause mortality, the deaths caused by respiratory disease and cardiovascular disease presents different space hot spots. For example, in 2015, Shanghai [4.62 (95% CI: 1.29–9.61) thousand], Taizhou [2.99 (95% CI: 1.77–4.19) thousand] and Nantong [2.88 (95% CI: 1.71–4.03) thousand] had the highest burden of cardiovascular disease, while Shanghai [2.82 (95% CI: 0.97–4.67) thousand], Chongqing [1.56 (95% CI: 1.05–2.08) thousand] and Chengdu [1.02 (95% CI: 0.68–1.35) thousand] had the highest burden of respiratory disease, and this difference lasted until 2018. It can be seen that Jiangsu Province (ranged from 27.72 thousand in 2016 to 40.92 thousand in 2017), Jiangsu Province (ranged from 22.33 thousand in 2016 to 33.32 thousand in 2017) and Sichuan Province (ranged from 5.12 thousand in 2015 to 6.75 thousand in 2017) are facing the most serious health burden of all-cause, cardiovascular and respiratory disease. Meanwhile, Fig. 4 shows the trend that the provincial-level health burden increased first and then decreased. At the national level, from 2015 to 2017, all-cause mortality increased by 44%, the cardiovascular increased mortality by 20%, and respiratory mortality increased by 28%. From 2017 to 2018, the all-cause, circulatory and respiratory mortality decreased by 15, 20, and 14%, respectively. Fig. S7 shows the spatial distribution pattern of all-cause mortality changes in 2015–2016, 2016–2017 and 2017–2018 in China. It can be seen that the health burden of the BTH region, Central Plains city belt and Yangtze-Huaihe River city belt increased significantly from 2015 to 2016 (> 200 people per city). From 2016 to 2017, the nationwide health burden generally increased, especially in the Central-Liaoning city belt, Shandong Peninsula, YRD region, Chengdu city and Wuhan city belt (> 1000 people per city). From 2017 to 2018, the burden of Central-Liaoning city belt, Shandong Peninsula, YRD region, Chengdu city and Wuhan city belt dropped significantly (> 1000 people per city).

### Ozone-related economic loss

**Fig. 5:**
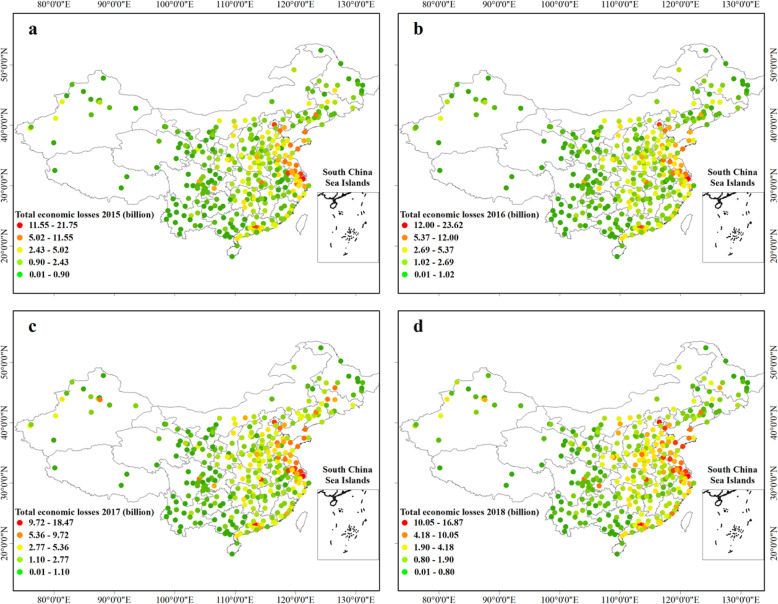
The city-specific economic losses of O3-related all-cause mortality in 334 Chinese cities in 2015 (**a**), 2016 (**b**), 2017 (**c**) and 2018 (**d**). The map was generated using the ArcMap 10.5 software, and the shape file were built-in resources of the software

**Fig. 6:**
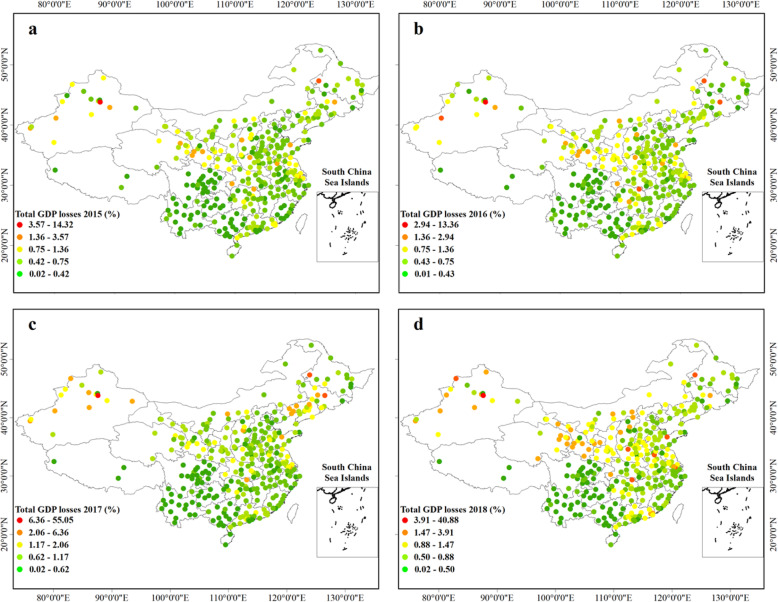
The city-specific GDP impact of O3-related all-cause mortality in 334 Chinese cities in 2015 (**a**), 2016 (**b**), 2017 (**c**) and 2018 (**d**). The map was generated using the ArcMap 10.5 software, and the shape file were built-in resources of the software

Figure 5 presents the spatial distribution of economic losses attributable to short-term O_3_ exposure in China during the period 2015–2018. ozone related all-cause deaths in China caused economic losses of 387.76 (95% CI: 195.99–904.50) billion, 413.92 (95% CI: 209.88–970.96) billion, 594.08 (95% CI: 303.34–1140.65) billion and 517.28 (95% CI: 263.76–1027.11) billion Yuan in 2015, 2016, 2017 and 2018, accounting for 0.52, 0.52, 0.69, 0.56% of the total GDP, respectively. Shanghai, Guangzhou and Beijing were the cities with the biggest economic losses. In addition, the relatively high economic losses still existed in cities on the BTH region, the YRD region, the PRD region, the Chengdu-Chongqing city belt and the Shandong peninsula (> 2.00 billion per city). However, the impact of premature death on GDP (%) presents a different distribution pattern than the economic burden mainly concentrated in densely populated areas (Fig. 6). Wujiaqu in Xinjiang (maximum 55.05% in 2017), Qitaihe (maximum 4.28% in 2017) in Heilongjiang, Liaoyuan (maximum 6.36% in 2017) in Jilin have the most serious GDP loss in China. It can be seen that most of these cities were located in the northwest and northeast of China.

Fig. S8 and S9 illustrate the economic losses from cardiovascular and respiratory disease due to short-term ozone exposure in China in 2015–2018. The cardiovascular mortality related to O_3_ caused economic losses of 230.42 (95% CI: 84.55–387.19) billion, 243.50 (95% CI: 86.88–412.20) billion, 358.03 (95% CI: 129.47–600.29) billion, 309.25 (95% CI: 111.58–520.00) billion in 2015, 2016, 2017, 2018 in China, respectively. Similar to the characteristics of all-cause economic losses, in 2015, 2016 and 2018, Beijing, Shanghai and Guangzhou had the largest cardiovascular economic burden, while in 2017, Guangzhou, Wuxi and Nantong had the largest economic cost. The respiratory mortality related to O_3_ caused economic losses of 67.00 (95% CI: 22.65–112.88) billion, 70.46 (95% CI: 24.32–118.37) billion, 93.31 (95% CI: 31.25–157.92) billion, 82.96 (95% CI: 28.67–139.45) billion in 2015, 2016, 2017, 2018 in China, respectively. Different from the distribution of the economic burden of all-causes death, Shanghai, Chongqing and Chengdu had the largest cardiovascular economic burden.

**Fig. 7:**
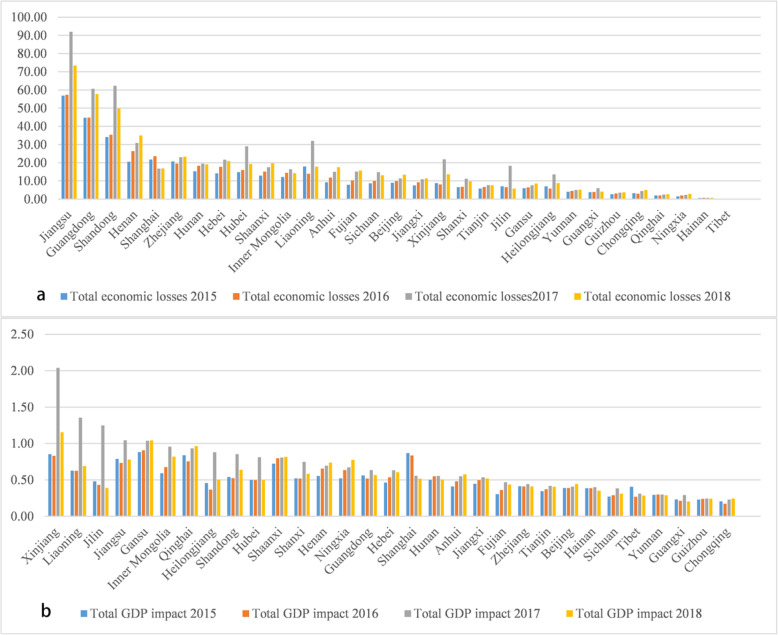
Provincial-level economic loss (billion) (**a**) and GDP impact (%) (**b**) due to premature death attributable to O3 short-term exposure during 2015–2018

Figure 7 and Table. S4, S5 illustrate the O_3_-related economic losses and GDP impacts in each province of China. It can be seen that Jiangsu, Guangdong, Shandong Province were facing the most severe economic burden caused by all-cause deaths, whereas Xinjiang, Liaoning, Jilin had the most massive GDP loss. Similarly, we could observe the trend of economic loss increasing first and then decreasing. From 2015 to 2017, the increase of ozone concentration intensified the national economic loss by 206.31 billion Yuan. From 2017 to 2018, the decrease of mortality saved the economic loss by 76.79 billion Yuan. The change of city-level economic loss is shown in Fig. S10.

## Discussion

As a strong oxidant, ozone is harmful to human health and agricultural production, in addition, as an important greenhouse gas, ozone plays an important role in global climate change [84, 85]. Due to its dual harm to health and climate, ozone has been paid close attention by environmental scientists and regulators in the past decades [86–88]. In China, economic development and urbanization have brought about serious environmental problems. Air pollutants have become an essential factor endangering the health of residents, and the government has begun to take intervention measures against pollutants [8, 89]. The previous control strategies always put priority on particulate matter and acid rain [90]. Actually, the concentration of particles in China has been decreasing year by year, but the problem of ozone pollution has become worse. Our study found that the national ozone concentration has increased significantly in mainland China from 2015 to 2018 and maintained at a high level, although the concentration of ozone in some regions decreased from 2017 to 2018. Especially from 2016 to 2017, cities in the BTH and YRD region, Xinjiang Province and Tibet Province increased by more than 50 μg/m^3^. In addition to the North China Plain, the YRD, the PRD, the Shandong Peninsula and Chengdu-Chongqing areas were facing high annual average concentration, we found that the ozone pollution in the Qinghai-Tibet Plateau and Gansu area was also severe, which was manifested in the high proportion of nonattainment days (even higher than North China Plain) that cannot be reflected by the annual average ozone concentration. Various reasons might explain the long-term nonattainment ozone concentration on the Qinghai-Tibet Plateau. One of them is because the precursor material from the long-distance transport is the basis of the formation of ozone in the Tibetan Plateau, and the unique strong near-surface convergence and upper layer divergence driven by elevated surface heating and low air density over the Qinghai-Tibet Plateau provides the driving force for this transport [91, 92]. As for Sichuan Basin, a large number of VOCs and NO_x_ were emitted, the topography of the surrounding the mountains and the unique atmospheric circulation generated a high concentration of ozone [93]. In other regions, such as the BTH region, the effect of long-distance transport is not apparent due to the high local emissions, while the transport in the adjacent regions aggravates the rise of local ozone concentration [94]. From 2006 to 2011, the emission rate of precursors in Beijing has actually decreased. However, the VOCs from nearby areas still make the surface ozone concentration rise [95]. In a previous research, the CMAQ model was used to evaluate the possible emission reduction measures, and it was found that if only the local emissions in Beijing were reduced (even 90%), it could not even meet the CNAAQS Grade II (160 μg/m^3^). Only by reducing the emissions in Beijing, Tianjin and Hebei simultaneously by 60 to 80% could the significant effect be achieved [96]. Therefore, the government needs to take specific preventive measures according to the unique causes of local ozone pollution in order to achieve the desired results.

Although there is a lack of national all-cause mortality studies, Wang’s study reported the number of disease-specific mortality caused by long-term ozone exposure in China from 2013 to 2017, with an average of 0.31 million premature mortality per year from cardiovascular and respiratory diseases [97]. In our study, an average of 0.22 million people died of cardiovascular and respiratory diseases caused by short-term ozone exposure every year, accounting for 70.96% of the long-term exposure related deaths reported by Wang [97]. This shows that China’s ozone burden is mainly caused by short-term exposure. Therefore, in China, we cannot ignore the health impacts caused by short-term exposure to O_3_. From 2015 to 2017, the national O_3_ exposure related health burden generally increased, especially in the North China Plain, Shandong Peninsula, Central-Liaoning, the YRD, the PRD and Chengdu-Chongqing region. This is consistent with the trend of ozone concentration, suggesting that the increase in health burden over the past 2015–2017 is probably due to the aggravation of ozone pollution. During 2017–2018, ozone pollution in priority areas such as the BTH region, the YRD, the PRD and the Shandong peninsula has been generally improved, and government intervention measures have avoided 40,000 premature deaths, suggesting that local policymakers have begun to pay attention to ozone pollution and formulate targeted intervention measures.

Due to the lack of nationwide short-term ozone health burden assessment studies in China, only a few studies were used for uncertainty analysis. Some scholars have comprehensively evaluated the health burden of long-term exposure to ozone in China, but the results show heterogeneity, which may be caused by the estimation of exposure, the selection of outcomes and the setting of thresholds. At present, most studies use chemical transport models or ground monitoring stations to estimate ozone exposure. In 2016, Madaniyazi combined two chemical transport models to estimate the ozone health burden in East China in 2030 [75]. The results show that the number of premature deaths without intervention ranges from 40,000 to 260,000. In 2018, Lin and Liu used the WRF-CMAQ model to estimate the number of COPD premature deaths due to ozone exposure in 2014 and 2015 respectively, and the results show that the value was 32.22–141.65 thousand and 55.34–80.28 thousand respectively [12, 19]. In 2019, Maji estimated that 74.2 thousand people died of long-term exposure to ozone in China in 2016 using ground monitoring station data [20]. In the same period, Liang estimated the avoidable all-cause mortality from short-term exposure to ozone in 2016, and the results showed that if the DMA8 dropped to CNAAQS Grade I (100 μg/m^3^), the premature death of 120 (95% CI: 67–160) thousand people could be avoided [21]. Theoretically, because air pollution monitoring stations are mainly distributed in city areas and densely populated areas, there is great uncertainty in the estimated value of the sites in the sparsely populated areas such as rural areas. For the chemical transport model, there are some limitations, such as the uncertainty of the input emission list and the coarse resolution of the model, which may lead to too high simulation results. Therefore, future research should focus on combining the advantages of the two methods to make mutual correction between the ground data and the model results. In addition, there is no definite conclusion about the choice of threshold. Madaniyazi et al. chose WHO AQG standard (100 μg/m^3^) as threshold [75], Lin et al. chose 37.6 ppb as threshold [19], Liu et al. and Maji et al. use 75.2 μg/m^3^ and 112 μg/m^3^ as threshold [12, 20], Yao et al. used 0 μg/m^3^ as threshold [30]. In this study, we chose zero as the threshold because studies conducted in China [15, 18, 98] and North America [99–101] found that a threshold did not exist in the acute effect of short-term O_3_ exposure.

For the economic burden, our study found that its spatiotemporal trend is almost the same as that of premature death, but the GDP loss presents different spatial characteristics. We found in northwest Xinjiang, Gan-Ning city belt, Guan Zhong city belt and Central-Liaoning city belt have a considerable loss of GDP, whose economic development lags behind that of BTH, YRD, PRD area. Severe pollution has been caused because the local government has neglected to protect the local environment in pursuit of rapid economic development. The decision-makers of these areas should simultaneously develop the economy considering the protection of environment, so as to achieve the coordinated development of both sides. In addition, uncertainty can also be introduced in estimating economic loss. In our study, ozone related all-cause deaths in China caused economic losses of 387.77 (95% CI: 195.99–904.50) to 594.08 (95% CI: 303.34–1140.65) billion Yuan during 2015–2018, accounting for 0.52 to 0.69% of the total GDP. The study of Xie et al. estimates that by 2030, the economic loss caused by ozone may range from 200 to 230 billion Dollars, accounting for 2.3 to 2.7% of GDP loss in that year [33]. This estimate is slightly higher than our studies because the outcome considers both mortality and morbidity. Maji et al. estimated that China’s ozone related premature death in 2016 caused an economic burden of 760 million Yuan, while Yao et al. pointed out that the economic loss due to ozone short-term exposure in 2017 was 49.6 billion Yuan [20, 30]. In addition to the types of health endpoints, another reason for the differences in economic valuation is the different valuation methods. For example, Liang used two valuation methods, VSL and AHC, to estimate the avoidable economic loss due to short-term ozone exposure in 2016, and the result showed that if the ozone concentration reduce to the expected value, the economic loss can be avoided by 36 and 64 billion Yuan respectively [21].

Moreover, the influence of exposure parameters on the results should not be neglected and should be considered in future research. We conducted a sensitivity analysis and calculated the percentage of the difference between the provinces with and without exposure parameters in the various health impacts, as shown in Table. S5 and Fig. S11. Overestimation mainly occurs in Guangdong (− 1242 to − 1500), Jiangsu (− 1564 to − 2315), which indicates that the actual daily exposure of residents tends to be lower due to the introduction of exposure parameters. Underestimation mainly occurs in Henan (1160 to − 1615), Xinjiang (663 to 1453), Gansu (616 to 755), which indicates that the actual daily exposure of residents tends to be higher. We can find that Eastern provinces are overestimated and Western provinces are underestimated, which reflects the exposure differences caused by the differences of living habits and environmental conditions between the East and the West of China (Fig. S11). Since this error was not considered in previous studies, the daily average concentration of monitoring stations was used to represent the actual daily exposure of residents, which may lead to the bias of the results, especially in some local studies [37, 75, 102]. Future research should not only consider the concentration of pollutants in the environment, but also refine the actual daily exposure to obtain more unbiased disease burden estimates.

However, there are several uncertainties. Firstly, using single permeability coefficient to simulate indoor exposure in this study may lead to deviation from the actual indoor exposure dose: In fact, there are few studies on permeability of ozone in China, and the existing research is conducted in single city [103–105], and there are not enough nationwide studies to show how these coefficients differ across regions. The data of single study [80] used in this paper may lead to the bias of indoor exposure dose. More importantly, there is still no effective exposure-response function simultaneous estimate indoor and outdoor exposure of ozone. Indoor-sources ozone exposure has not been genuinely estimated in this study, which is also a problem to be solved in future studies. Second, in this study, we assume that ozone is an independent pollutant. In other words, we do not consider the interaction caused by other pollutants (such as PM_2.5_) when estimating the health effects of ozone. In fact, past studies have shown that the negative effects of ozone can be enhanced when multiple pollutants coexist [106–109]. Estimating the health burden caused by ozone separately may overlook this synergistic effect and result in underestimation. Thirdly, due to the unavailability of mortality rate data, we can only distribute the monthly baseline mortality rate evenly to every day after getting the monthly mortality baseline rate at the city level (Table. S6), which ignored the original temporal trend of incidence rates, leading to bias in the estimation of actual health impact. Previous studies have shown that haze events can cause fluctuations in mortality: light, medium and heavy dust-haze days were associated with increased mortality of 3.4, 6.8 and 10.4% respectively [110]. Future studies should try to get the daily mortality rates of each city as accurately as possible to get the actual fluctuations to calculate more reliable results. Fourth, although we know that individual exposure has high space-time variation, this study used uniform monitoring station data to represent the exposure level and static population data, which may affect the estimation accuracy of this study. Fifth, as mentioned above, we believe that there is no threshold in the acute exposure reaction curve of ozone, so the minimum ozone concentration is set to zero, but the reality of ground-level ozone falling to zero is almost non-existent. In GBD 2019, the minimum risk exposure level was specified as a uniform distribution between 29.1 and 35.7 ppb (57.1 and 70.1 μg/m3) [111]. Moreover, Anenberg et al. found that worldwide, anthropogenic emissions accounted for only 37% of ozone attributable asthma related impacts [112]. This indicates that the economic burden calculated in this study include the ozone concentration below the achievable minimum concentration and that emitted by non-anthropogenic, which has no practical significance for the government’s financial investment. Finally, although the calculation is simplified by using excess risk instead of traditional attributable fraction in Formula 1, the best approximation can be achieved only when the pollutant concentration is relatively low (about 10 μg/m^3^). With the relative increase of ozone concentration, the overestimation of the result will gradually increase. For example, in 2015, Beijing, the city with the highest ozone concentration (201.30 μg/m^3^), will have the most serious overestimation. According to the all-cause mortality exposure response coefficient *RR* = 1.0037 for Beijing, the all-cause premature death will be overestimated by 7.4%.

Although there are some limitations in this study, it is the first time to analyze the spatiotemporal distribution of the health and economic burden caused by short-term exposure to ozone in China, and to make up for the shortcomings of previous studies by using refined methods to make the estimation results more accurate. The results of the study provide policy makers with the changing trend of ozone concentration and burden in China, and provide enlightening information for the formulation of new priority areas for prevention and control. Although the uncertainty affects the estimation accuracy, this study presents a reliable overall temporal trend and spatial hot spots.

## Conclusions

To our knowledge, this is the first study in China evaluating the cause-specific premature death and economic loss attributable to short-term O_3_ exposure at the national level during 2015–2018 and adopted the exposure parameter weighting method to compensate for the estimation bias caused by the ignorance of the spatial discrepancies of parameters employed in the exposure-response function. In summary, our study provided evidence that short-term exposure to O_3_ results in substantial health and economic burden in China which is cannot be ignored. The overall health and economic burden across the country are on the decline, at least until 2018. However, policy makers need to strengthen the supervision of underdeveloped regions and realize the coordinated development of economy and health. Meanwhile, in areas with high pollution burden, the government should shift the focus of policy intervention as soon as possible and formulate energy-saving and emission reduction measures for ozone to reduce the heavy burden.

## Supplementary Information

**Additional file 1 Figure S1.** The city-specific baseline mortality in 334 Chinese cities in 2015, 2016, 2017 and 2018. **Figure S2.** The city-specific VSL in 334 Chinese cities in 2015, 2016, 2017 and 2018. **Figure S3.** The city-specific permanent population in 334 Chinese cities in 2015, 2016, 2017 and 2018. **Figure S4.** Changes in 90th daily maximum 8-h O_3_ concentrations in 334 Chinese cities from 2015 to 2016, 2016 to 2017, 2017 to 2018. **Figure S5.** The city-specific O_3_-realted cardiovascular mortality in 334 Chinese cities in 2015, 2016, 2017 and 2018. **Figure S6.** The city-specific O_3_-realted respiratory mortality in 334 Chinese cities in 2015, 2016, 2017 and 2018. **Figure S7.** Changes in all-cause mortality in 334 Chinese cities from 2015 to 2016, 2016 to 2017, 2017 to 2018. **Figure S8.** The city-specific economic losses of O_3_-related cardiovascular mortality in 334 Chinese cities in 2015, 2016, 2017 and 2018. **Figure S9.** The city-specific economic losses of O_3_-related respiratory mortality in 334 Chinese cities in 2015, 2016, 2017 and 2018. **Figure S10.** Changes in economic loss of all-cause mortality in 334 Chinese cities from 2015 to 2016, 2016 to 2017, 2017 to 2018. **Figure S11.** Differences of health impacts with and without exposure factors at city-level in 2015, 2016, 2017 and 2018. **Table S1.** Provincial-level statistics of air pollution related exposure parameters. **Table S2.** Provincial level O_3_ attributable health impacts during 2015–2018 (thousand). **Table S3.** Provincial level O_3_ attributable economic loss during 2015–2018 (billion Yuan). **Table S4.** Provincial level O_3_ attributable GDP impact during 2015–2018 (%). **Table S5.** Differences of health impacts with and without exposure factors at provincial and national level (person) / (%). **Table S6.** Monthly proportion of the deaths in Chinese province (%).

## Data Availability

The pollutant data are available from the China National Environmental Monitoring Center (http://113.108.142.147:20035/emcpublish/).The city-specific socio-economic data was derived from the National Statistical Yearbook in each Province or the National Economic and Social Development Bulletins of each city, including permanent residents, per capita GDP, Price index, mortality rate (available at: https://data.cnki.net/). Exposure parameters data were extracted from Exposure Factors Handbook of Chinese Population.

## Abbreviations

VSL: Value of statistical life
GDP: Gross domestic product
VOCs: Volatile organic compounds
NOx: Nitrogen oxides
BTH: Beijing-Tianjin-Hebei region
YRD: Yangtze River Delta
PRD: Pearl River Delta
COPD: Chronic obstructive pulmonary diseases
WTP: Willingness to pay
DMA8: Daily ambient maximum 8-h average
CEERHAPS: Chinese Environmental Exposure-Related Human Activity Patterns Survey
WHO: World Health Organization
ERFs: Exposure-response functions
I/O ratio: indoor/outdoor ratio
HCA: Human capital approach
OECD: Organization for Economic Co-operation and Development
CPI: Consumer price index
CNAAQS: China National Ambient Air Quality Standards
CMAQ: Community Multiscale Air Quality

## References

[1] USEPA (2006). Air quality criteria for ozone and related photochemical oxidants. vol. 1: EPA Research Triangle Park, NC.

[2] USEPA (2013). Integrated science assessment for ozone and related photochemical oxidants.

[3] Sakizadeh M, Mohamed MM. Application of spatial analysis to investigate contribution of VOCs to photochemical ozone creation. Environ Sci Pollut Res Int. 2020;27:10459–71. 10.1007/s11356-020-07628-4 31939025

[4] Bernard SM, Samet JM, Grambsch A, Ebi KL, Romieu I (2001). The potential impacts of climate variability and change on air pollution-related health effects in the United States. Environ Health Perspect.

[5] Penrod A, Zhang Y, Wang K, Wu S-Y, Leung LR (2014). Impacts of future climate and emission changes on U.S. air quality. Atmos Environ.

[6] Madronich S, Shao M, Wilson SR, Solomon KR, Longstreth JD, Tang XY (2015). Changes in air quality and tropospheric composition due to depletion of stratospheric ozone and interactions with changing climate: implications for human and environmental health. Photochem Photobiol Sci.

[7] Wu R, Xie S (2017). Spatial distribution of ozone formation in China derived from emissions of Speciated volatile organic compounds. Environ Sci Technol.

[8] Chen Z, Wang JN, Ma GX, Zhang YS (2013). China tackles the health effects of air pollution. Lancet.

[9] Wang Y, Li W, Gao W, Liu Z, Tian S, Shen R, et al. Trends in particulate matter and its chemical compositions in China from 2013–2017. Sci Chin Earth Sci. 2019;62(12):1857–71.

[10] Liu H, Wang XM, Pang JM, He KB (2013). Feasibility and difficulties of China's new air quality standard compliance: PRD case of PM2.5 and ozone from 2010 to 2025. Atmos Chem Phys.

[11] Wang Y, Gao W, Wang S, Song T, Gong Z, Ji D, et al. Contrasting trends of PM2.5 and surface ozone concentrations in China from 2013 to 2017. Natl Sci Rev. 2020;7(8):1331–9. 10.1093/nsr/nwaa032 PMC8288972 34692161

[12] Liu H, Liu S, Xue B, Lv Z, Meng Z, Yang X, et al. Ground-level ozone pollution and its health impacts in China. Atmos Environ. 2018;173:223–30.

[13] Lu X, Hong J, Zhang L, Cooper OR, Schultz MG, Xu X, et al. Severe surface ozone pollution in China: a global perspective. Environ Sci Technol Lett. 2018;5(8):487–94.

[14] Yin P, Chen R, Wang L, Meng X, Liu C, Niu Y, et al. Ambient ozone pollution and daily mortality: a Nationwide study in 272 Chinese cities. Environ Health Perspect. 2017;125(11):117006. 10.1289/EHP1849 PMC5947936 29212061

[15] Chen K, Zhou L, Chen X, Bi J, Kinney P (2017). Acute effect of ozone exposure on daily mortality in seven cities of Jiangsu Province, China: no clear evidence for threshold. Environ Res.

[16] Shang Y, Sun Z, Cao J, Wang X, Zhong L, Bi X, et al. Systematic review of Chinese studies of short-term exposure to air pollution and daily mortality. Environ Int. 2013;54:100–11. 10.1016/j.envint.2013.01.010 23434817

[17] Tao Y, Huang W, Huang X, Zhong L, Lu SE, Li Y, et al. Estimated acute effects of ambient ozone and nitrogen dioxide on mortality in the Pearl River Delta of southern China. Environ Health Perspect. 2012;120(3):393–8. 10.1289/ehp.1103715 PMC3295344 22157208

[18] Wong TW, Vichit-Vadakan N, Kan H, Qian Z (2008). Public health and air pollution in Asia (PAPA): a multicity study of short-term effects of air pollution on mortality. Environ Health Perspect.

[19] Lin Y, Jiang F, Zhao J, Zhu G, He X, Ma X, et al. Impacts of O3 on premature mortality and crop yield loss across China. Atmos Environ. 2018;194:41–7.

[20] Maji KJ, Ye WF, Arora M, Nagendra SMS (2019). Ozone pollution in Chinese cities: assessment of seasonal variation, health effects and economic burden. Environ Pollut.

[21] Liang S, Li X, Teng Y, Fu H, Chen L, Mao J, et al. Estimation of health and economic benefits based on ozone exposure level with high spatial-temporal resolution by fusing satellite and station observations. Environ Pollut. 2019;255(Pt 2):113267. 10.1016/j.envpol.2019.113267 31574391

[22] Li T, Yan M, Ma W, Ban J, Liu T, Lin H, et al. Short-term effects of multiple ozone metrics on daily mortality in a megacity of China. Environ Sci Pollut Res. 2015;22(11):8738–46. 10.1007/s11356-014-4055-5 25572272

[23] Liu T, Li TT, Zhang YH, Xu YJ, Lao XQ, Rutherford S, et al. The short-term effect of ambient ozone on mortality is modified by temperature in Guangzhou, China. Atmos Environ. 2013;76:59–67.

[24] Pascal M, Wagner V, Chatignoux E, Falq G, Corso M, Myriam B, et al. Ozone and short-term mortality in nine French cities: influence of temperature and season. Atmos Environ. 2012;62:566–72.

[25] Lim C, Hayes R, Ahn J, Shao Y, Silverman D, Jones R, et al. Long-term Exposure to Ozone and Cause-Specific Mortality Risk in the U.S. Am J Respir Crit Care Med. 2019;200(8):1022–31. 10.1164/rccm.201806-1161OC PMC6794108 31051079

[26] Bell M, McDermott A, Zeger S, Samet J, Dominici F (2004). Ozone and short-term mortality in 95 US Urban communities, 1987-2000. JAMA.

[27] Wang N, Mengersen K, Tong S, Kimlin M, Zhou M, Wang L, et al. Short-term association between ambient air pollution and lung cancer mortality. Environ Res. 2019;179:108748. 10.1016/j.envres.2019.108748 31561053

[28] Zhang M, Song Y, Cai X (2007). A health-based assessment of particulate air pollution in urban areas of Beijing in 2000–2004. Sci Total Environ.

[29] Yang Y, Luo L, Song C, Yin H, Yang J. Spatiotemporal Assessment of PM2.5-Related Economic Losses from Health Impacts during 2014–2016 in China. Int J Environ Res Public Health. 2018;15(6):1278. 10.3390/ijerph15061278 PMC6024949 29914184

[30] Yao M, Wu G, Zhao X, Zhang J (2020). Estimating health burden and economic loss attributable to short-term exposure to multiple air pollutants in China. Environ Res.

[31] Maji KJ, Ye WF, Arora M, Shiva Nagendra SM (2018). PM2.5-related health and economic loss assessment for 338 Chinese cities. Environ Int.

[32] Lu X, Yao T, Fung JCH, Lin C (2016). Estimation of health and economic costs of air pollution over the Pearl River Delta region in China. Sci Total Environ.

[33] Xie Y, Dai H, Zhang Y, Wu Y, Hanaoka T, Masui T (2019). Comparison of health and economic impacts of PM2.5 and ozone pollution in China. Environ Int.

[34] Konz JJ, Lisi K, Friebele E, Dixon DA (1989). Exposure Factors Handbook.

[35] Men C, Liu R, Xu F, Wang Q, Guo L, Shen Z (2018). Pollution characteristics, risk assessment, and source apportionment of heavy metals in road dust in Beijing, China. Sci Total Environ.

[36] Gao W, Cao D, Wang Y, Wu J, Wang Y, Wang Y, et al. External exposure to short- and medium-chain chlorinated Paraffins for the general population in Beijing, China. Environ Sci Technol. 2018;52(1):32–9. 10.1021/acs.est.7b04657 29190090

[37] Wang JD, Wang SX, Voorhees AS, Zhao B, Jang C, Jiang JK, et al. Assessment of short-term PM2.5-related mortality due to different emission sources in the Yangtze River Delta, China. Atmos Environ. 2015;123:440–8.

[38] Hu J, Huang L, Chen M, Liao H, Zhang H, Wang S, et al. Premature mortality attributable to particulate matter in China: source contributions and responses to reductions. Environ Sci Technol. 2017;51(17):9950–9. 10.1021/acs.est.7b03193 28787143

[39] Wang Z, Wu T, Duan X, Wang S, Zhang W, Wu X, et al. Research on inhalation rate exposure factors of Chinese residents in environmental health risk assessment, vol. 22; 2009. p. 1171–5.

[40] Zou B, You J, Lin Y, Duan X, Zhao X, Fang X, et al. Air pollution intervention and life-saving effect in China. Environ Int. 2019;125:529–41. 10.1016/j.envint.2018.10.045 30612707

[41] Zhang YL, Cao F (2015). Fine particulate matter (PM 2.5) in China at a city level. Sci Rep.

[42] Zhao S, Yu Y, Yin D, He J, Liu N, Qu J, et al. Annual and diurnal variations of gaseous and particulate pollutants in 31 provincial capital cities based on in situ air quality monitoring data from China National Environmental Monitoring Center. Environ Int. 2016;86:92–106. 10.1016/j.envint.2015.11.003 26562560

[43] Song C, He J, Wu L, Jin T, Chen X, Li R, et al. Health burden attributable to ambient PM2. 5 in China. Environ Pollut. 2017;223:575–86. 10.1016/j.envpol.2017.01.060 28169071

[44] Koleski K (2017). The 13th five-year plan. US-China Economic and Security Review Commission*,* Staff Research Report, USA.

[45] Shi W, Sun Q, Du P, Tang S, Chen C, Sun Z, et al. Modification effects of temperature on the ozone-mortality relationship: a nationwide multicounty study in China. Environ Sci Technol. 2020;54(5):2859–68. 10.1021/acs.est.9b05978 32022552

[46] Vicedo-Cabrera AM, Sera F, Liu C, Armstrong B, Milojevic A, Guo Y, et al. Short term association between ozone and mortality: global two stage time series study in 406 locations in 20 countries. BMJ. 2020;368:m108. 10.1136/bmj.m108 PMC7190035 32041707

[47] Mokoena KK, Ethan CJ, Yu Y, Shale K, Liu F (2019). Ambient air pollution and respiratory mortality in Xi’an, China: a time-series analysis. Respir Res.

[48] Yim SHL, Wang M, Gu Y, Yang Y, Dong G, Li Q (2019). Effect of urbanization on ozone and resultant health effects in the Pearl River Delta region of China. J Geophys Res.

[49] NHCPRC (2017). China Health and Family Planning Statistics Yearbook 2018: National Health Commission of the People's Republic of China.

[50] Duan X, Zhao X, Wang B, Chen Y, Cao S (2015). Highlights of the Chinese exposure factors handbook (adults).

[51] Yin P, Brauer M, Cohen A, Burnett R, Liu J, Liu Y, et al. Long-term fine particulate matter exposure and nonaccidental and cause-specific mortality in a large National Cohort of Chinese Men. Environ Health Perspect. 2017;125(11):117002. 10.1289/EHP1673 PMC5947939 29116930

[52] Lim C, Hayes R, Ahn J, Shao Y, Silverman D, Jones R, et al. Association between long-term exposure to ambient air pollution and diabetes mortality in the US. Environ Res. 2018;165:330–6. 10.1016/j.envres.2018.04.011 PMC5999582 29778967

[53] Zhou M, Liu Y, Wang L, Kuang X, Xu X, Kan H (2013). Particulate Air Pollution and Mortality in a Cohort of Chinese Men. Environ Pollut (Barking, Essex : 1987).

[54] Chen X, Wang X, Huang J-J, Zhang L, Song F, Mao H-J, et al. Nonmalignant respiratory mortality and long-term exposure to PM10 and SO2: A 12-year cohort study in northern China. Environ Pollut (Barking, Essex : 1987). 2017;231:761–7. 10.1016/j.envpol.2017.08.085 28865381

[55] Chen X, Zhang L, Huang J-J, Song F, Zhang L-P, Qian Z-M, et al. Long-term exposure to urban air pollution and lung cancer mortality: a 12-year cohort study in northern China. Sci Total Environ. 2016;571:855–61. 10.1016/j.scitotenv.2016.07.064 27425436

[56] Li T, Zhang Y, Wang J, Xu D, Zhaoxue Y, Chen H, et al. All-cause mortality risk associated with long-term exposure to ambient PM2·5 in China: a cohort study. Lancet Public Health. 2018;3:e470–7. 10.1016/S2468-2667(18)30144-0 30314593

[57] Guo B, Chen F, Deng Y, Zhang H, Qiao X, Qiao Z, et al. Using rush hour and daytime exposure indicators to estimate the short-term mortality effects of air pollution: a case study in the Sichuan Basin, China. Environ Pollut. 2018;242(Pt B):1291–8. 10.1016/j.envpol.2018.08.028 30121483

[58] Brook R, Sun Z, Brook J, Zhao X, Ruan Y, Yan J, et al. Extreme air pollution conditions adversely affect blood pressure and insulin resistance: the air pollution and Cardiometabolic disease study. Hypertension. 2015;67(1):77–85. 10.1161/HYPERTENSIONAHA.115.06237 PMC4830086 26573709

[59] Li T, Yan M, Sun Q, Anderson G (2017). Mortality risks from a spectrum of causes associated with wide-ranging exposure to fine particulate matter: a case-crossover study in Beijing, China. Environ Int.

[60] Shah A, Lee K, McAllister D, Hunter A, Nair H, Whiteley W, et al. Short term exposure to air pollution and stroke: Systematic review and meta-analysis. BMJ (Clinical research ed). 2015;350:h1295. 10.1136/bmj.h1295 PMC4373601 25810496

[61] Cai Y, Zhang B, Ke W, Feng B, Lin H, Xiao J, et al. Associations of Short-Term and Long-Term Exposure to Ambient Air Pollutants With Hypertension: A Systematic Review and Meta-Analysis. Hypertension. 2016;68(1):62–70. 10.1161/HYPERTENSIONAHA.116.07218 27245182

[62] Lu F, Xu D, Cheng Y, Dong S, Guo C, Jiang X, et al. Systematic review and meta-analysis of the adverse health effects of ambient PM2.5 and PM10 pollution in the Chinese population. Environ Res. 2014;136C:196–204. 10.1016/j.envres.2014.06.029 25460637

[63] Zhang S, Li G, Tian L, Guo Q, Pan X (2016). Short-term exposure to air pollution and morbidity of COPD and asthma in east Asian area: a systematic review and meta-analysis. Environ Res.

[64] Thi Trang Nhung N, Amini H, Schindler C, Joss M, Minh Dien T, Probst-Hensch N, et al. Short-term association between ambient air pollution and pneumonia in children: A systematic review and meta-analysis of time-series and case-crossover studies. Environ Pollut. 2017;230:1000–8. 10.1016/j.envpol.2017.07.063 28763933

[65] Tian Y, Liu H, Liang T, Xiang X, Li M, Juan J, et al. Ambient air pollution and daily hospital admissions: a nationwide study in 218 Chinese cities. Environ Pollut. 2018;242(Pt B):1042–9. 10.1016/j.envpol.2018.07.116 30096542

[66] Roberts S, Arseneault L, Barratt B, Beevers S, Danese A, Odgers CL, et al. Exploration of NO2 and PM2.5 air pollution and mental health problems using high-resolution data in London-based children from a UK longitudinal cohort study. Psychiatry Res. 2019;272:8–17. 10.1016/j.psychres.2018.12.050 PMC6401205 30576995

[67] Guo P. Maternal exposure to gaseous ambient air pollutants increases the risk of preterm birth in the Pearl River Delta, China 2014-2017. Sci Total Environ. 2019;671:959–70.

[68] Maji KJ, Arora M, Dikshit AK (2017). Burden of disease attributed to ambient PM2.5 and PM10 exposure in 190 cities in China. Environ Sci Pollut Res.

[69] Hubbell BJ, Fann N, Levy JI (2009). Methodological considerations in developing local-scale health impact assessments: balancing national, regional, and local data. Air Qual Atmos Health.

[70] Pascal M, Corso M, Chanel O, Declercq C, Badaloni C, Cesaroni G, et al. Assessing the public health impacts of urban air pollution in 25 European cities: results of the Aphekom project. Sci Total Environ. 2013;449:390–400. 10.1016/j.scitotenv.2013.01.077 23454700

[71] Lelieveld J, Barlas C, Giannadaki D, Pozzer A (2013). Model calculated global, regional and megacity premature mortality due to air pollution. Atmos Chem Phys.

[72] Yin H, Pizzol M, Xu L (2017). External costs of PM2.5 pollution in Beijing, China: uncertainty analysis of multiple health impacts and costs. Environ Pollut.

[73] Knowlton K, Rosenthal JE, Hogrefe C, Lynn B, Gaffin S, Goldberg R, et al. Assessing ozone-related health impacts under a changing climate. Environ Health Perspect. 2004;112(15):1557–63. 10.1289/ehp.7163 PMC1247621 15531442

[74] Madaniyazi L, Nagashima T, Guo Y, Yu W, Tong S (2015). Projecting fine particulate matter-related mortality in East China. Environ Sci Technol.

[75] Madaniyazi L, Nagashima T, Guo Y, Pan X, Tong S (2016). Projecting ozone-related mortality in East China. Environ Int.

[76] Zheng S, Pozzer A, Cao CX, Lelieveld J (2015). Long-term (2001–2012) concentrations of fine particulate matter (PM<sub>2.5</sub>) and the impact on human health in Beijing, China. Atmos Chem Phys.

[77] WHO (2007). Air quality guidelines: Global update 2005. Particulate matter, ozone, nitrogen dioxide and sulfur dioxide. Indian J Med Res.

[78] Burnett RT, Pope CA III, Ezzati M, Olives C, Lim SS, Mehta S, et al. An integrated risk function for estimating the global burden of disease attributable to ambient fine particulate matter exposure. Environ Health Perspect. 2014;122(4):397–403. 10.1289/ehp.1307049 PMC3984213 24518036

[79] Chen H, Yun L, Su Q, Cheng L (2015). Spatial variation of multiple air pollutants and their potential contributions to all-cause, respiratory, and cardiovascular mortality across China in. Atmos Environ.

[80] Yang Z, Shen J, Gao Z (2018). Ventilation and air quality in student dormitories in China: a case study during summer in Nanjing. Int J Environ Res Public Health.

[81] Giannadaki D, Giannakis E, Pozzer A, Lelieveld J (2018). Estimating health and economic benefits of reductions in air pollution from agriculture. Sci Total Environ.

[82] Xie XX (2011). The value of health: applications of choice experiment approach and Urban air pollution control strategy.

[83] OECD (2014). The cost of air pollution: health impacts of road transport: OECD.

[84] Koike K, Inoue G, Fukuda T (1999). Explosion hazard of gaseous ozone. J Chem Eng Jpn.

[85] Singh BP, Kumar A, Singh D, Punia M, Kumar K, Jain VK (2014). An assessment of ozone levels, UV radiation and their occupational health hazard estimation during photocopying operation. J Hazard Mater.

[86] Malig B, Pearson D, Chang B, Broadwin R, Basu R, Green R, et al. A Time-Stratified Case Crossover Study of Ambient Ozone Exposure and Emergency Department Visits for Specific Respiratory Diagnoses in California (2005-2008). Environ Health Perspect. 2015:124(6):745–53. 10.1289/ehp.1409495 PMC4892911 26647366

[87] Colette A, Granier C, Hodnebrog Ø, Jakobs H, Maurizi A, Nyiri A, et al. Bessagnet B, amp *et al*: future air quality in Europe: a multi-model assessment of projected exposure to ozone. Atmos Chem Phys. 2012;12(21):10613–30.

[88] Watanabe M, Yamaguchi M, Matsumura H, Kohno Y, Koike T, Izuta T (2011). A case study of risk assessment of ozone impact on forest tree species in Japan. Asian J Atmospheric Environ.

[89] Li X, Qiao Y, Zhu J, Shi L, Wang Y (2017). The “APEC blue” endeavor: causal effects of air pollution regulation on air quality in China. J Clean Prod.

[90] Wang T, Xue L, Brimblecombe P, Lam YF, Li L, Zhang L (2017). Ozone pollution in China: a review of concentrations, meteorological influences, chemical precursors, and effects. Sci Total Environ.

[91] Wang T, Wong HLA, Tang J, Ding A, Wu WS, Zhang XC. On the origin of surface ozone and reactive nitrogen observed at a remote mountain site in the northeastern Qinghai-Tibetan Plateau, western China. J Geophys Res. 2006;111(D8):D08303.

[92] Li J, Wang Z, Akimoto H, Tang J, Uno I. Modeling of the impacts of China's anthropogenic pollutants on the surface ozone summer maximum on the northern Tibetan Plateau. Geophys Res Lett. 2009:36(24):L24802.

[93] Zhao S, Yu Y, Qin D, Yin D, Dong L, He J (2019). Analyses of regional pollution and transportation of PM2.5 and ozone in the city clusters of Sichuan Basin, China. Atmos Pollut Res.

[94] Fu J, Dong X, Gao Y, Wong D, Lam Y (2012). Sensitivity and linearity analysis of ozone in East Asia: The effects of domestic emission and intercontinental transport. J Air Waste Manag Assoc (1995).

[95] Zhang Q, Yuan B, Shao M, Wang X, Lu S, Lu K, et al. Variations of ground-level O-3 and its precursors in Beijing in summertime between 2005 and 2011. Atmos Chem Phys. 2014;14:6089–101.

[96] Xing J, Wang S, Jang C, Zhu Y, Hao J. Nonlinear response of ozone to precursor emission changes in China: a modeling study using response surface methodology. Atmos Chem Phys. 2011;11(10):5027–44.

[97] Wang Y, Wild O, Chen X, Wu Q, Gao M, Chen H, et al. Health impacts of long-term ozone exposure in China over 2013-2017. Environ Int. 2020;144:106030. 10.1016/j.envint.2020.106030 32798800

[98] Yang C, Yang H, Guo S, Wang Z, Xu X, Duan X, et al. Alternative ozone metrics and daily mortality in Suzhou: the China air pollution and health effects study (CAPES). Sci Total Environ. 2012;426:83–9. 10.1016/j.scitotenv.2012.03.036 22521098

[99] Peng R, Samoli E, Pham L, Dominici F, Touloumi G, Ramsay T, et al. Acute effects of ambient ozone on mortality in Europe and North America: results from the APHENA study. Air Qual Atmosphere Health. 2013;6:445–53. 10.1007/s11869-012-0180-9 PMC3668792 23734168

[100] Katsouyanni K, Samet J, Anderson H, Atkinson R, Le Tertre A, Medina S, et al. Air pollution and health: A European and North American approach (APHENA). Res Rep (Health Effects Institute). 2009;142:5–90. 20073322

[101] Bell M, Peng R, Dominici F (2006). The exposure–response curve for ozone and risk of mortality and the adequacy of current ozone regulations. Environ Health Perspect.

[102] Fan FY, Lei YL, Li L (2019). Health damage assessment of particulate matter pollution in Jing-Jin-Ji region of China. Environ Sci Pollut Res.

[103] Feng Y, Wen S, Wang X, Sheng G, He Q, Tang J, et al. Indoor and outdoor carbonyl compounds in the hotel ballrooms in Guangzhou, China. Atmos Environ. 2004;38(1):103–12.

[104] Tang J, Chan CY, Wang X, Chan LY, Sheng G, Fu J (2005). Volatile organic compounds in a multi-storey shopping mall in Guangzhou, South China. Atmos Environ.

[105] Du X, Liu J (2009). Relationship between outdoor and indoor ozone pollution concentration. Transact Tianjin Univ.

[106] Fakhri AA, Ilic LM, Wellenius GA, Urch B, Silverman F, Gold DR, et al. Autonomic effects of controlled fine particulate exposure in young healthy adults: effect modification by ozone. Environ Health Perspect. 2009;117(8):1287–92. 10.1289/ehp.0900541 PMC2721874 19672410

[107] Gleason JA, Bielory L, Fagliano JA (2014). Associations between ozone, PM2.5, and four pollen types on emergency department pediatric asthma events during the warm season in New Jersey: a case-crossover study. Environ Res.

[108] Jeanjean M, Bind M-A, Roux J, Ongagna J-C, de Sèze J, Bard D, et al. Ozone, NO2 and PM10 are associated with the occurrence of multiple sclerosis relapses. Evidence from seasonal multi-pollutant analyses. Environ Res. 2018;163:43–52. 10.1016/j.envres.2018.01.040 PMC5886008 29426027

[109] Jung C-R, Lin Y-T, Hwang B-F. Ozone, particulate matter, and newly diagnosed Alzheimer's disease: a population-based cohort study in Taiwan. JAD. 2014;44(2):573–84. 10.3233/JAD-140855 25310992

[110] Liu T, Zhang YH, Xu YJ, Lin HL, Xu XJ, Luo Y, et al. The effects of dust–haze on mortality are modified by seasons and individual characteristics in Guangzhou, China. Environ Pollut. 2014;187:116–23. 10.1016/j.envpol.2013.12.027 24477104

[111] Murray CJL, Aravkin AY, Zheng P, Abbafati C, Abbas KM, Abbasi-Kangevari M, et al. Global burden of 87 risk factors in 204 countries and territories, 1990-2019: a systematic analysis for the global burden of disease study 2019. Lancet. 2020;396(10258):1223–49. 10.1016/S0140-6736(20)30752-2 PMC7566194 33069327

[112] Anenberg S, Henze D, Tinney V, Kinney P, Raich W, Fann N, et al. Estimates of the Global Burden of Ambient PM2.5, Ozone, and NO2 on Asthma Incidence and Emergency Room Visits. Environ Health Perspect. 2018;126:107004. 10.1289/EHP3766 PMC6371661 30392403

