# Supplementary material for: Spatiotemporal assessment of health burden and economic losses attributable to short-term exposure to ground-level ozone during 2015–2018 in China
Source: BMC Public Health. 2021 Jun 5;21:1069. doi: 10.1186/s12889-021-10751-7 (PMC8178864; doi:10.1186/s12889-021-10751-7)
Supplement: Supplementary file 1 — Additional file 1 Figure S1. The city-specific baseline mortality in 334 Chinese cities in 2015, 2016, 2017 and 2018. Figure S2. The city-specific VSL in 334 Chinese cities in 2015, 2016, 2017 and 2018. Figure S3. The city-specific permanent population in 334 Chinese cities in 2015, 2016, 2017 and 2018. Figure S4. Changes in 90th daily maximum 8-h O3 concentrations in 334 Chinese cities from 2015 to 2016, 2016 to 2017, 2017 to 2018. Figure S5. The city-specific O3-realted cardiovascular mortality in 334 Chinese cities in 2015, 2016, 2017 and 2018. Figure S6. The city-specific O3-realted respiratory mortality in 334 Chinese cities in 2015, 2016, 2017 and 2018. Figure S7. Changes in all-cause mortality in 334 Chinese cities from 2015 to 2016, 2016 to 2017, 2017 to 2018. Figure S8. The city-specific economic losses of O3-related cardiovascular mortality in 334 Chinese cities in 2015, 2016, 2017 and 2018. Figure S9. The city-specific economic losses of O3-related respiratory mortality in 334 Chinese cities in 2015, 2016, 2017 and 2018. Figure S10. Changes in economic loss of all-cause mortality in 334 Chinese cities from 2015 to 2016, 2016 to 2017, 2017 to 2018. Figure S11. Differences of health impacts with and without exposure factors at city-level in 2015, 2016, 2017 and 2018. Table S1. Provincial-level statistics of air pollution related exposure parameters. Table S2. Provincial level O3 attributable health impacts during 2015–2018 (thousand). Table S3. Provincial level O3 attributable economic loss during 2015–2018 (billion Yuan). Table S4. Provincial level O3 attributable GDP impact during 2015–2018 (%). Table S5. Differences of health impacts with and without exposure factors at provincial and national level (person) / (%). Table S6. Monthly proportion of the deaths in Chinese province (%). [file 12889_2021_10751_MOESM1_ESM.docx]

**Supplementary material**

#
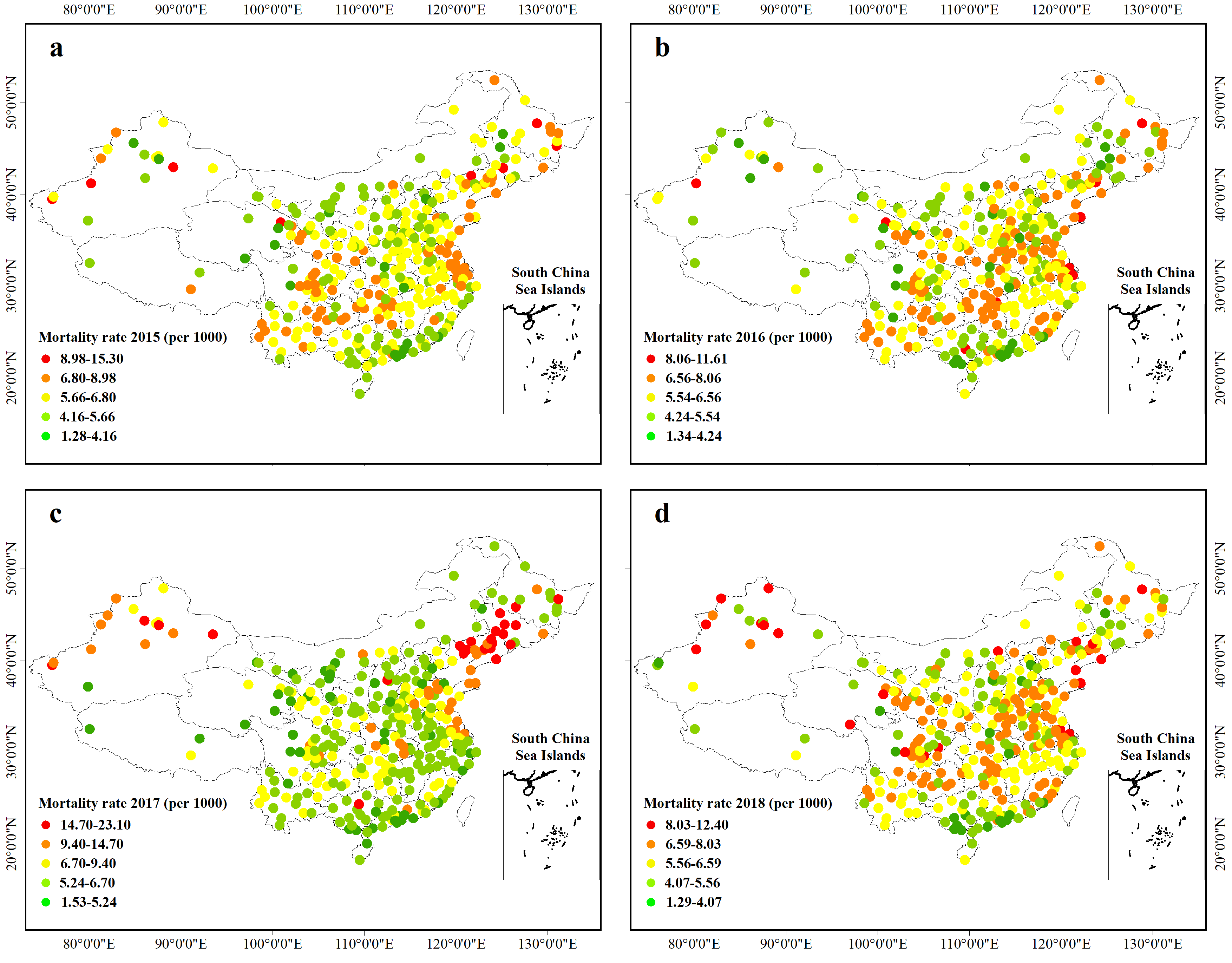
Figure. S1 The city-specific baseline mortality in 334 Chinese cities in 2015 (a), 2016 (b), 2017 (c) and 2018 (d). The map was generated using the ArcMap 10.5 software, and the shape file were built-in resources of the software.

#
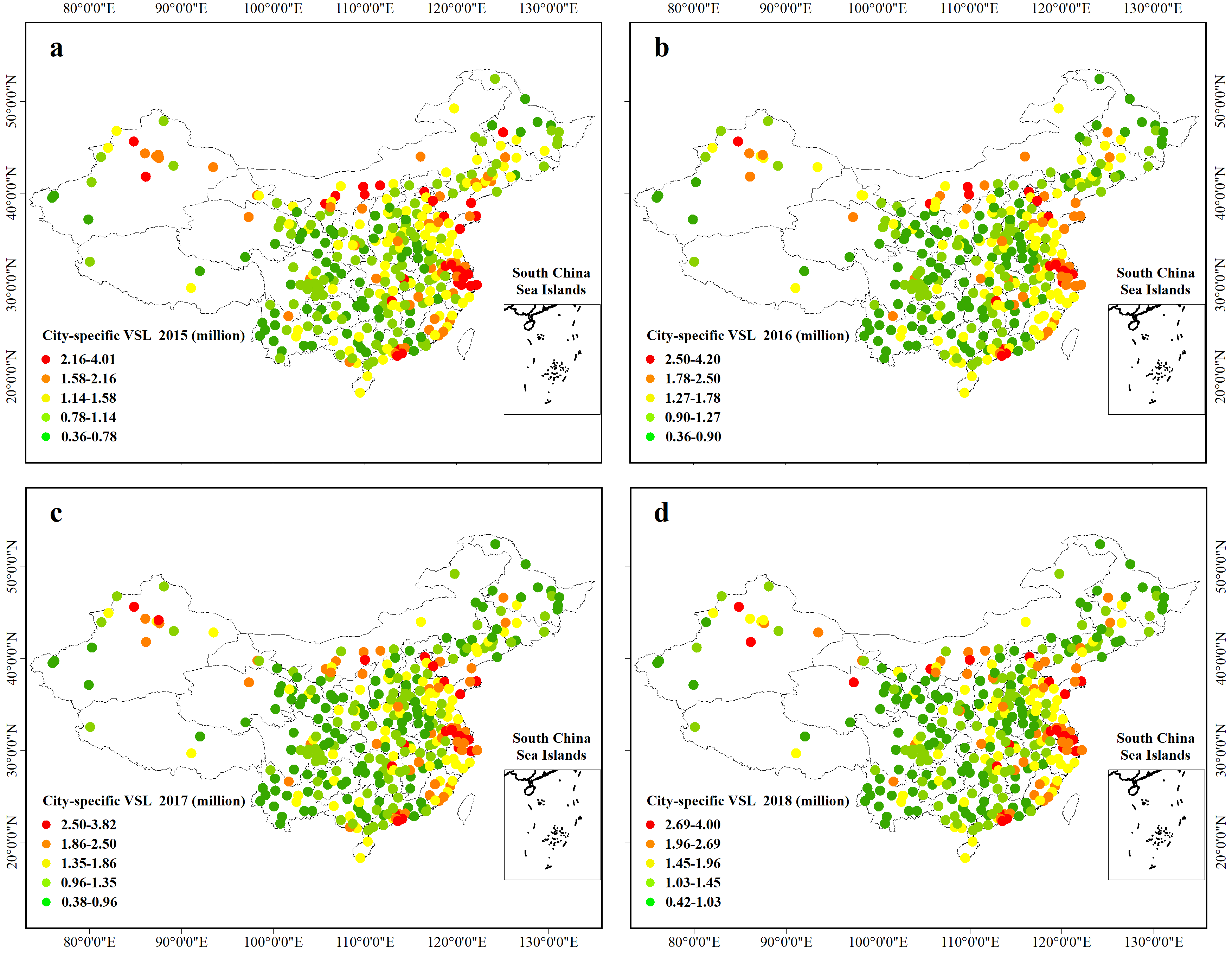
Figure. S2 The city-specific VSL in 334 Chinese cities in 2015 (a), 2016 (b), 2017 (c) and 2018 (d). The map was generated using the ArcMap 10.5 software, and the shape file were built-in resources of the software.

# Figure. S3 The city-specific permanent population in 334 Chinese cities in 2015 (a), 2016 (b), 2017 (c) and 2018 (d). The map was generated using the ArcMap 10.5 software, and the shape file were built-in resources of the software.

#
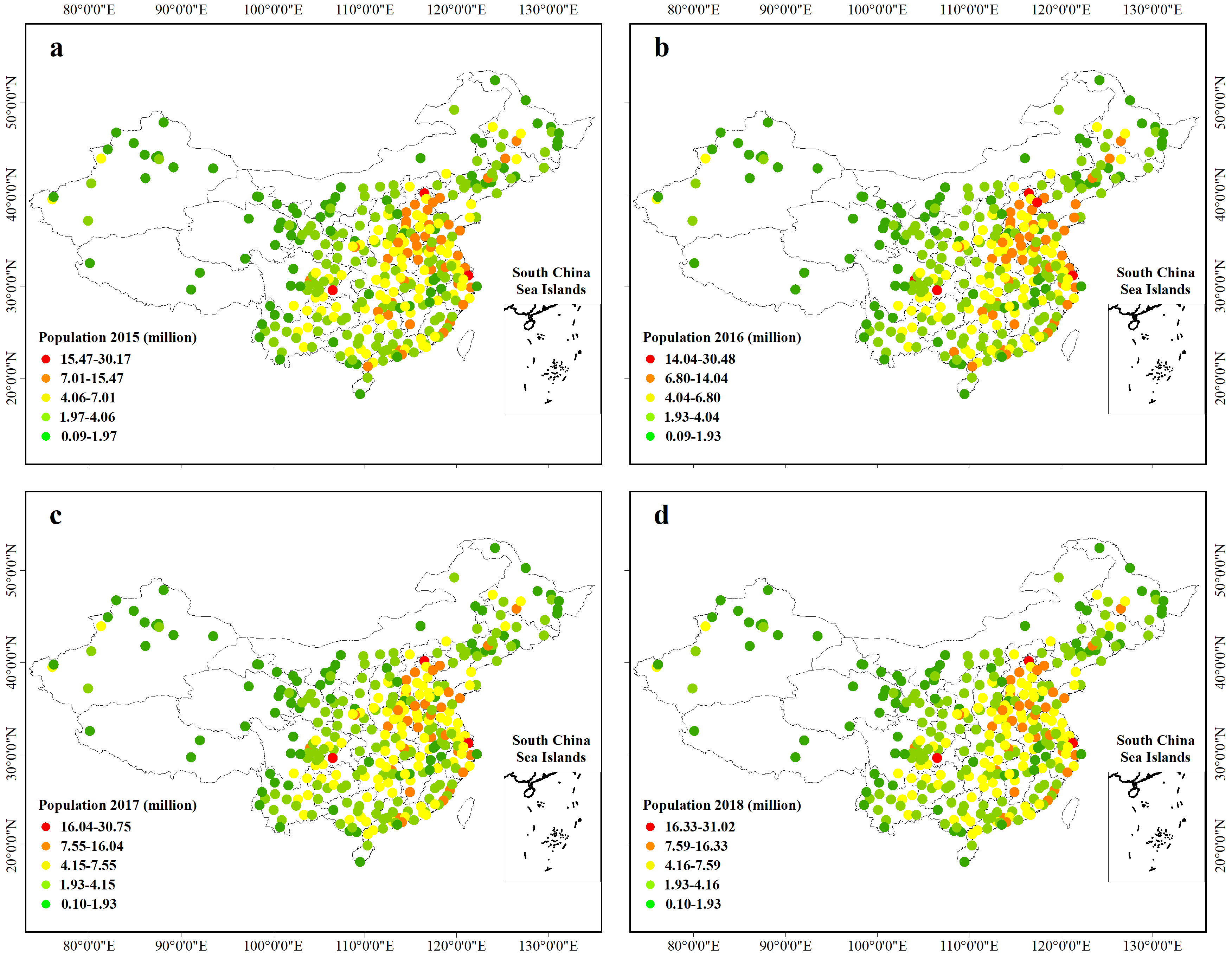


#
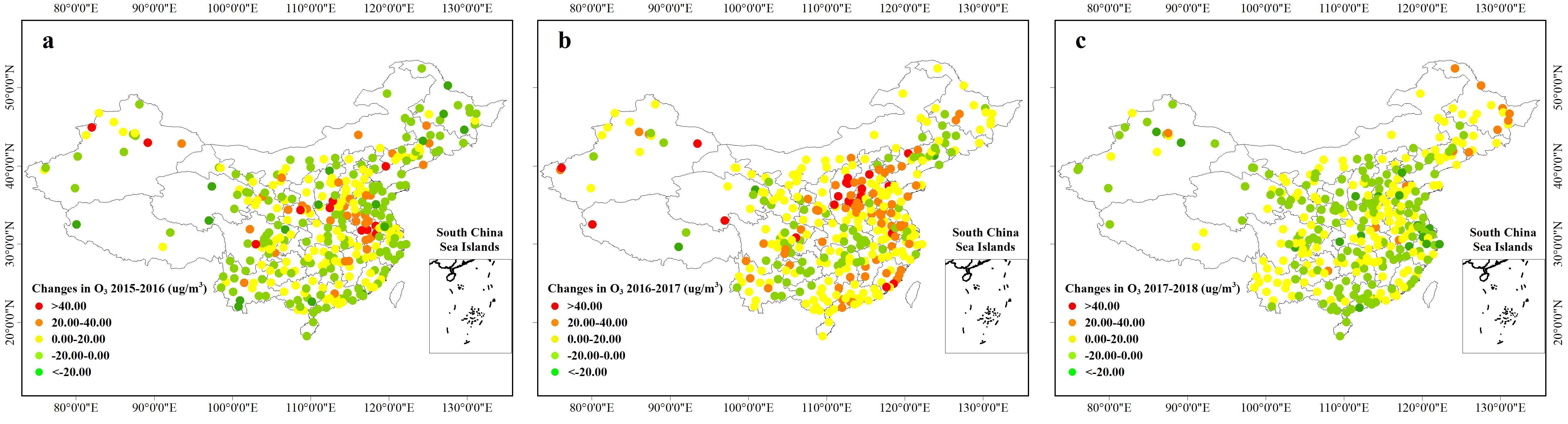
Figure. S4 Changes in 90^th^ daily maximum 8-h O_3_ concentrations in 334 Chinese cities from 2015 to 2016 (a), 2016 to 2017 (b), 2017 to 2018 (c). The map was generated using the ArcMap 10.5 software, and the shape file were built-in resources of the software.

#
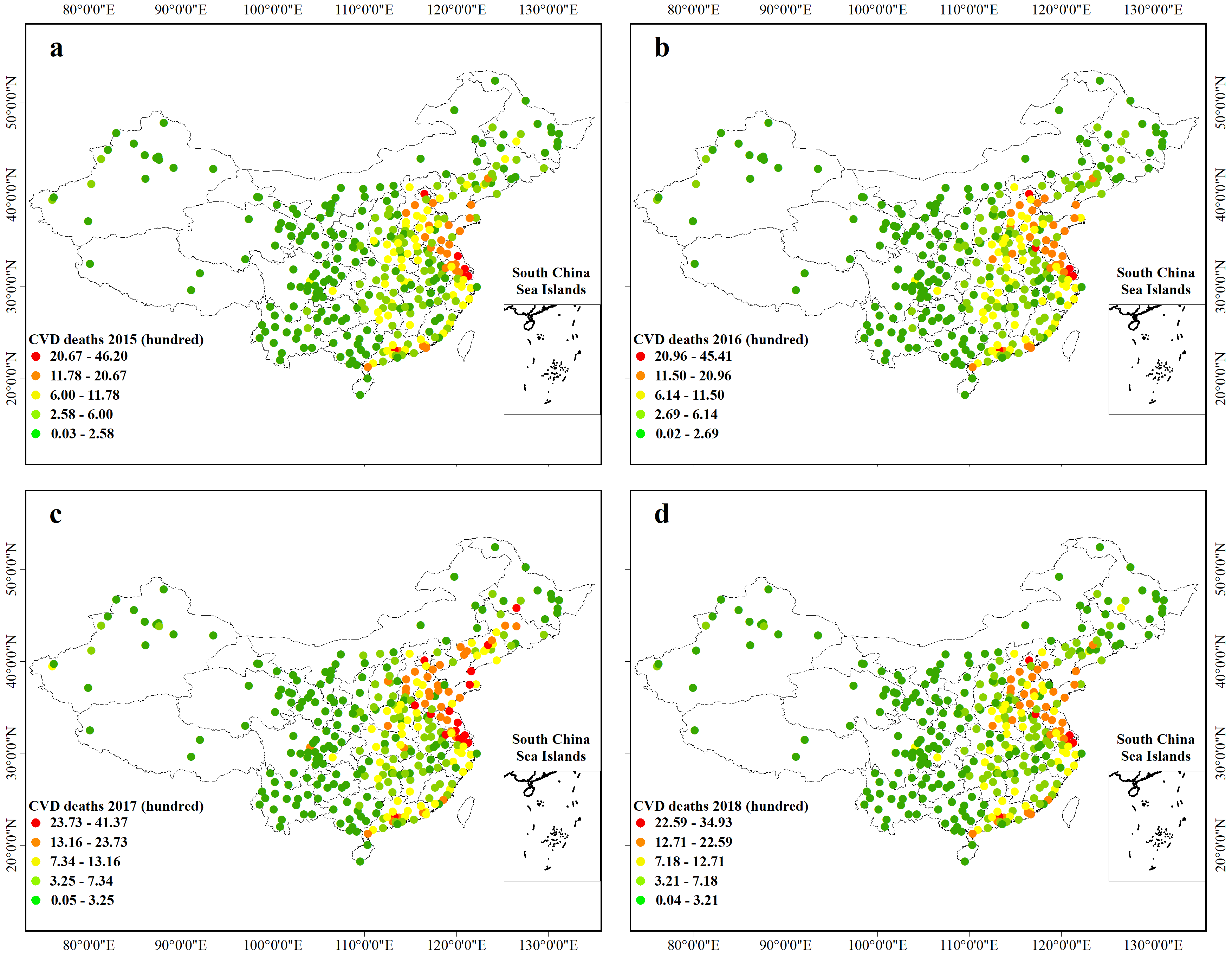
Figure. S5 The city-specific O3-realted cardiovascular mortality in 334 Chinese cities in 2015 (a), 2016 (b), 2017 (c) and 2018 (d). The map was generated using the ArcMap 10.5 software, and the shape file were built-in resources of the software.

#
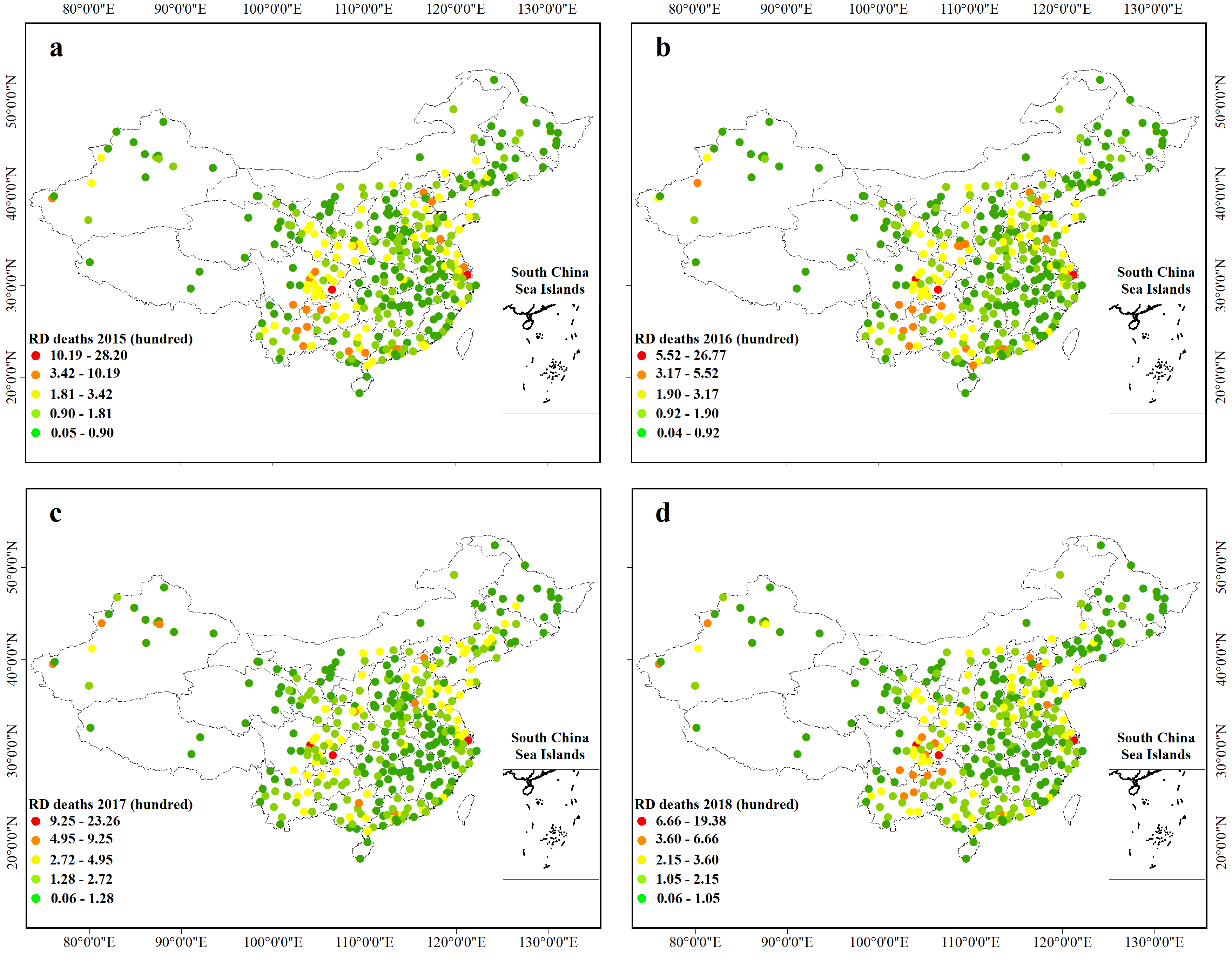
Figure. S6 The city-specific O3-realted respiratory mortality in 334 Chinese cities in 2015 (a), 2016 (b), 2017 (c) and 2018 (d). The map was generated using the ArcMap 10.5 software, and the shape file were built-in resources of the software.

# Figure. S7 Changes in all-cause mortality in 334 Chinese cities from 2015 to 2016 (a), 2016 to 2017 (b), 2017 to 2018 (c). The map was generated using the ArcMap 10.5 software, and the shape file were built-in resources of the software.


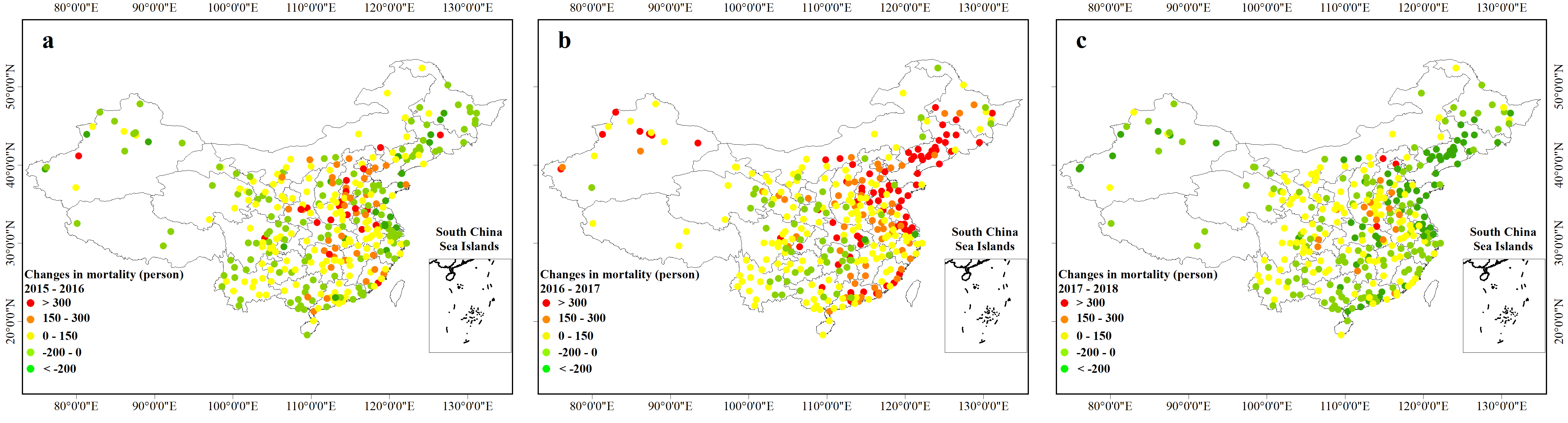


#
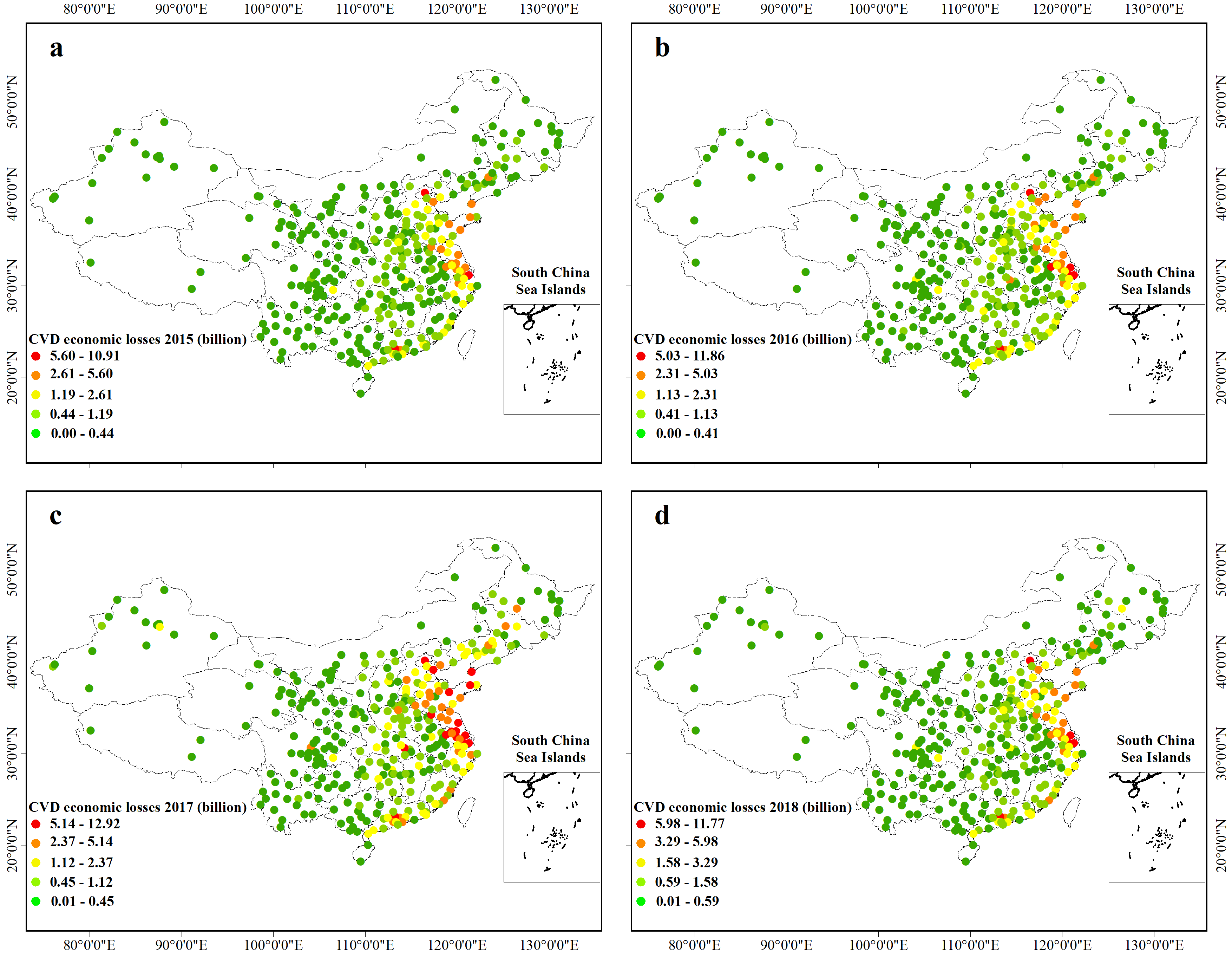
Figure. S8 The city-specific economic losses of O_3_-related cardiovascular mortality in 334 Chinese cities in 2015 (a), 2016 (b), 2017 (c) and 2018 (d). The map was generated using the ArcMap 10.5 software, and the shape file were built-in resources of the software.

#
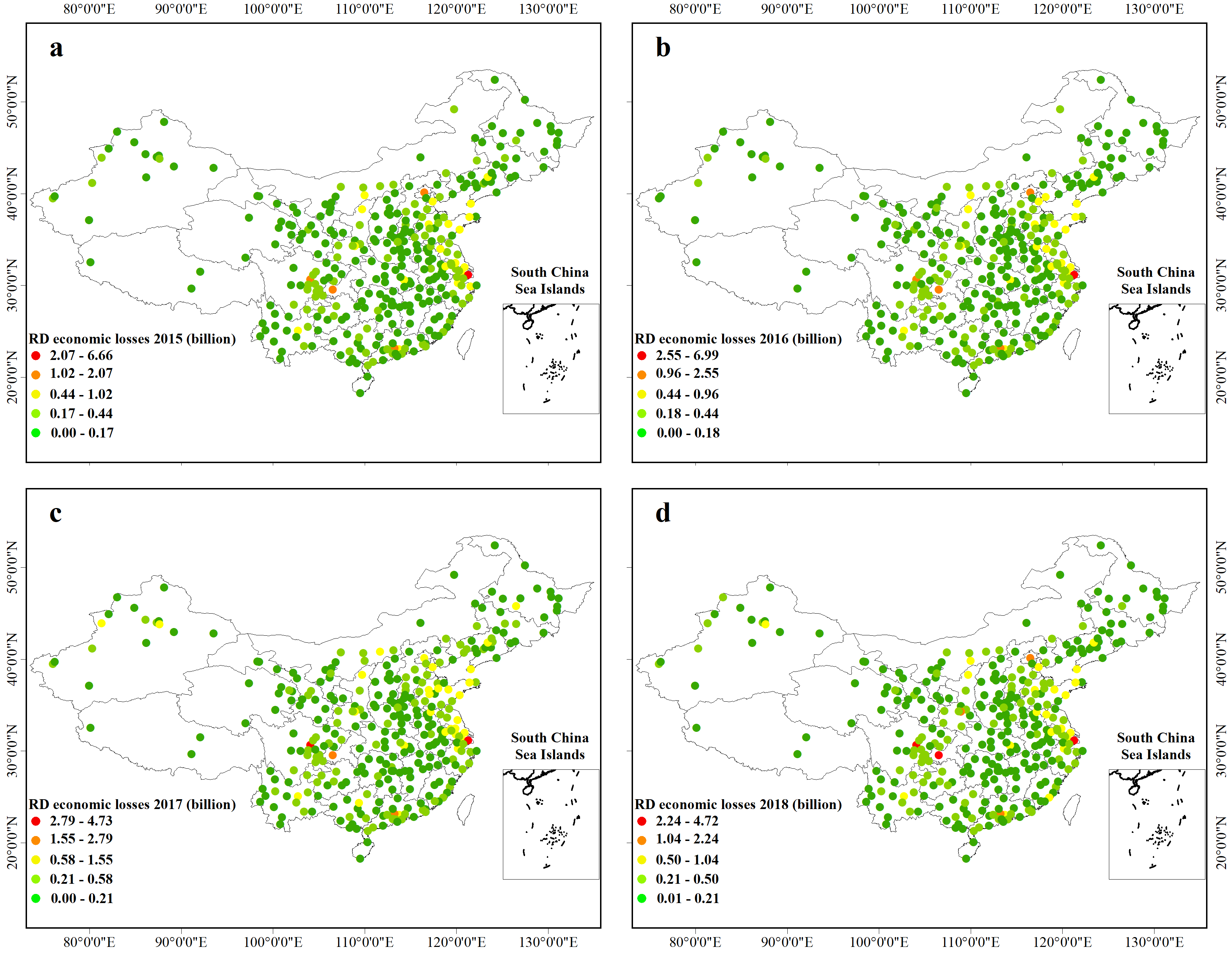
Figure. S9 The city-specific economic losses of O_3_-related respiratory mortality in 334 Chinese cities in 2015 (a), 2016 (b), 2017 (c) and 2018 (d). The map was generated using the ArcMap 10.5 software, and the shape file were built-in resources of the software.

#
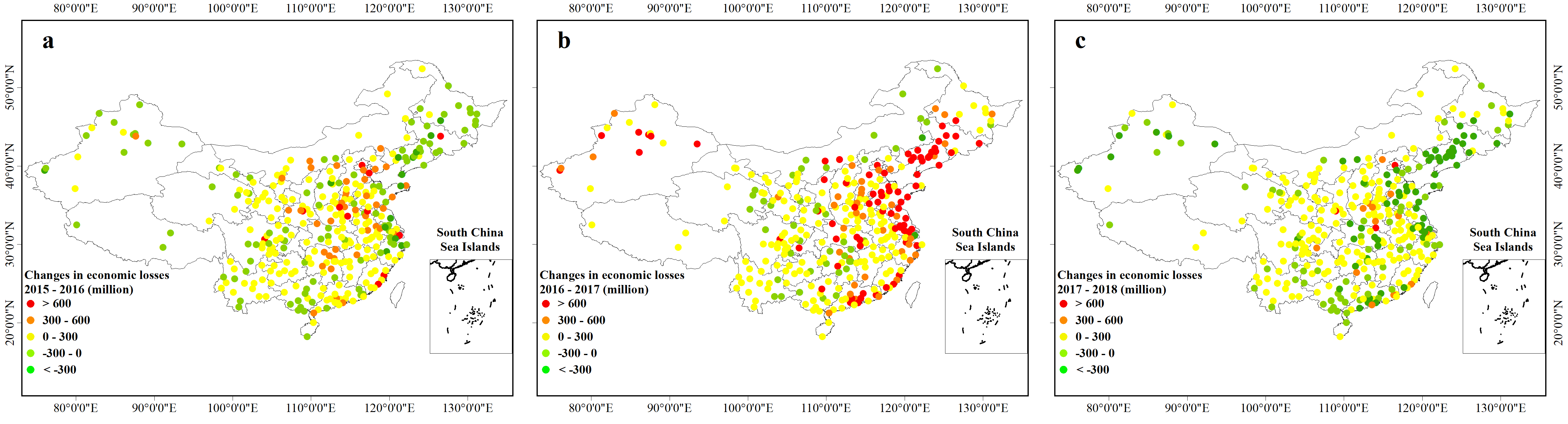
Figure. S10 Changes in economic loss of all-cause mortality in 334 Chinese cities from 2015 to 2016 (a), 2016 to 2017 (b), 2017 to 2018 (c). The map was generated using the ArcMap 10.5 software, and the shape file were built-in resources of the software.

# Figure. S11 Differences of health impacts with and without exposure factors at city-level in 2015 (a), 2016 (b), 2017 (c) and 2018 (d). The map was generated using the ArcMap 10.5 software, and the shape file were built-in resources of the software.
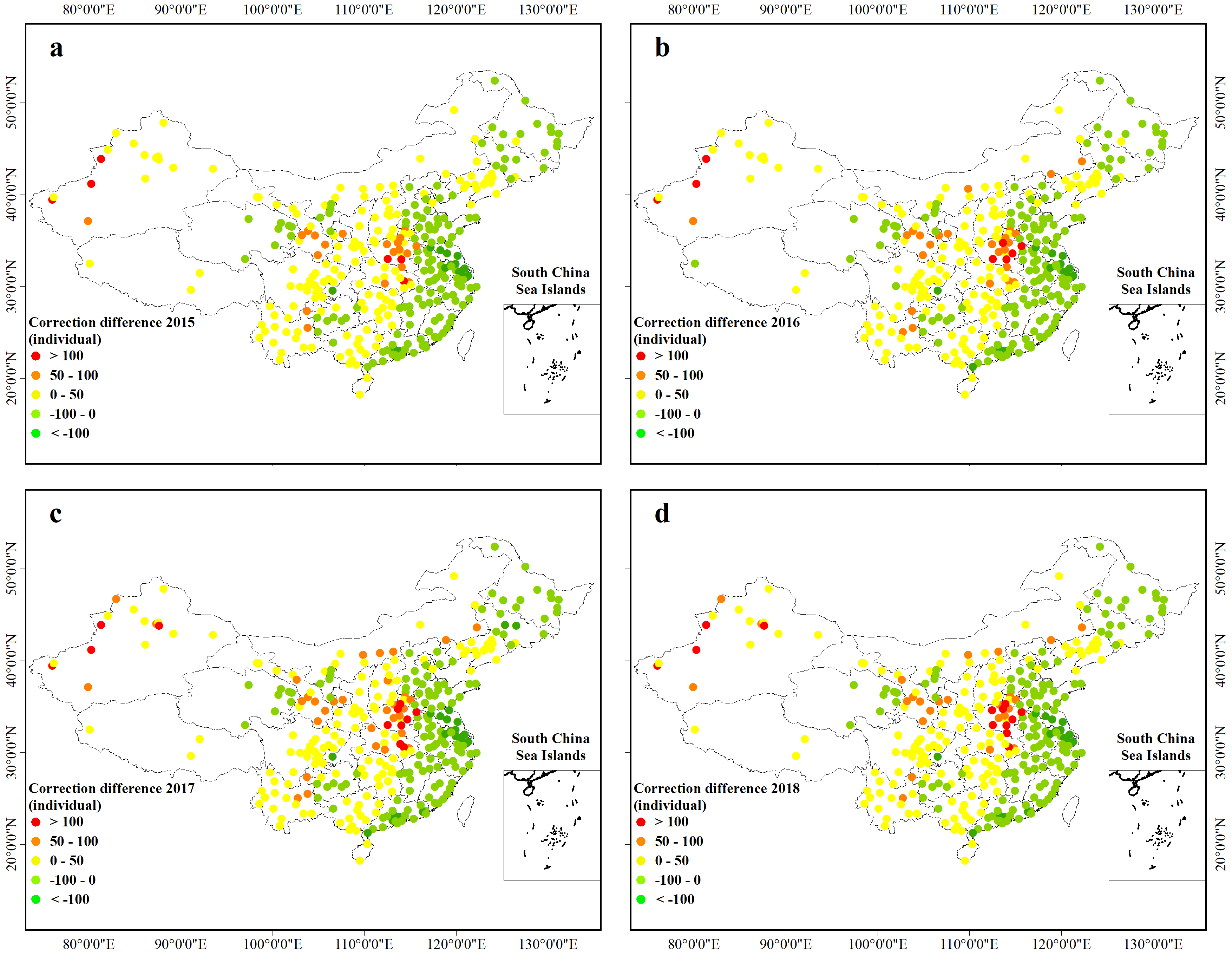


# Table.S1 Provincial-level statistics of air pollution related exposure parameters

| Provinces | Time spent outdoors(hour/day) | | | | Inhalation rates(m^3^/day) |
| --- | --- | --- | --- | --- | --- |
|  | Spring | Autumn | Summer | Winter |  |
| Anhui | 3.43 | 3.43 | 3.85 | 2.98 | 15.4 |
| Beijing | 3.28 | 3.28 | 4.00 | 2.38 | 16.1 |
| Fujian | 3.00 | 3.00 | 3.48 | 2.88 | 15.5 |
| Gansu | 5.50 | 5.50 | 7.15 | 3.28 | 15.6 |
| Guangdong | 3.50 | 3.50 | 4.00 | 3.00 | 15.0 |
| Guangxi | 4.67 | 4.67 | 5.82 | 3.85 | 15.1 |
| Guizhou | 3.98 | 3.98 | 4.52 | 2.50 | 15.6 |
| Hainan | 5.50 | 5.50 | 6.00 | 5.00 | 15.3 |
| Hebei | 3.00 | 3.00 | 4.08 | 2.00 | 16.0 |
| Henan | 5.00 | 5.00 | 6.00 | 3.83 | 15.6 |
| Heilongjiang | 3.50 | 3.50 | 5.00 | 0.95 | 16.0 |
| Hubei | 4.50 | 4.50 | 5.00 | 3.65 | 15.8 |
| Hunan | 3.67 | 3.67 | 4.00 | 2.67 | 15.8 |
| Jilin | 2.22 | 2.22 | 3.67 | 1.00 | 16.2 |
| Jiangsu | 3.00 | 3.00 | 3.00 | 2.28 | 15.4 |
| Jiangxi | 3.90 | 3.90 | 4.15 | 3.15 | 15.2 |
| Liaoning | 3.35 | 3.35 | 4.00 | 1.50 | 16.3 |
| Inner Mongolia | 3.77 | 3.77 | 5.83 | 2.10 | 16.3 |
| Ningxia | 2.85 | 2.85 | 3.35 | 2.00 | 16.0 |
| Qinghai | 2.57 | 2.57 | 4.00 | 2.00 | 16.0 |
| Shandong | 2.73 | 2.73 | 3.65 | 1.83 | 16.1 |
| Shanxi | 3.83 | 3.83 | 5.00 | 2.00 | 16.1 |
| Shaanxi | 4.72 | 4.72 | 5.43 | 3.17 | 15.1 |
| Shanghai | 2.57 | 2.57 | 2.82 | 2.03 | 15.8 |
| Sichuan | 4.22 | 4.22 | 4.50 | 3.57 | 15.4 |
| Tianjin | 3.00 | 3.00 | 4.07 | 2.00 | 16.2 |
| Tibet | 6.00 | 6.00 | 5.83 | 4.72 | 15.3 |
| Xinjiang | 4.77 | 4.77 | 6.00 | 3.12 | 16.3 |
| Yunnan | 5.43 | 5.43 | 6.00 | 4.50 | 15.5 |
| Zhejiang | 3.28 | 3.28 | 3.17 | 2.77 | 15.6 |
| Chongqing | 3.33 | 3.33 | 4.00 | 2.33 | 14.8 |
| National | 3.72 | 3.72 | 4.33 | 2.53 | 15.7 |

# Table.S2 Provincial level O_3_ attributable health impacts during 2015 – 2018 (thousand)

| Province | All-cause mortality | | | | Card-mortality | | | | Respir-mortality | | | |
| --- | --- | --- | --- | --- | --- | --- | --- | --- | --- | --- | --- | --- |
|  | 2015 | 2016 | 2017 | 2018 | 2015 | 2016 | 2017 | 2018 | 2015 | 2016 | 2017 | 2018 |
| Anhui | 9.46 | 11.31 | 13.41 | 14.29 | 4.58 | 5.51 | 6.55 | 6.98 | 0.85 | 1.00 | 1.18 | 1.25 |
| Beijing | 3.72 | 3.80 | 4.05 | 4.45 | 2.80 | 2.87 | 3.08 | 3.39 | 0.55 | 0.55 | 0.55 | 0.60 |
| Fujian | 4.91 | 5.98 | 8.07 | 7.68 | 3.69 | 4.51 | 6.15 | 5.85 | 0.73 | 0.86 | 1.10 | 1.04 |
| Gansu | 8.52 | 8.89 | 10.13 | 10.47 | 1.67 | 1.76 | 2.04 | 2.11 | 1.84 | 1.93 | 2.14 | 2.21 |
| Guangdong | 28.05 | 27.21 | 34.49 | 31.62 | 19.40 | 18.83 | 24.13 | 22.12 | 4.10 | 3.85 | 4.60 | 4.22 |
| Guangxi | 4.06 | 3.95 | 5.14 | 3.79 | 1.56 | 1.54 | 2.04 | 1.50 | 2.59 | 2.53 | 3.20 | 2.36 |
| Guizhou | 3.11 | 3.25 | 3.41 | 3.50 | 1.20 | 1.27 | 1.35 | 1.39 | 1.98 | 2.08 | 2.12 | 2.18 |
| Hainan | 0.42 | 0.43 | 0.45 | 0.41 | 0.32 | 0.32 | 0.35 | 0.31 | 0.06 | 0.06 | 0.06 | 0.06 |
| Hebei | 12.74 | 14.89 | 17.78 | 16.86 | 9.59 | 11.22 | 13.54 | 12.84 | 1.90 | 2.14 | 2.42 | 2.29 |
| Henan | 20.26 | 24.19 | 26.21 | 28.36 | 9.81 | 11.78 | 12.80 | 13.85 | 1.82 | 2.15 | 2.30 | 2.49 |
| Heilongjiang | 6.71 | 5.22 | 10.92 | 6.97 | 3.25 | 2.54 | 5.33 | 3.40 | 0.60 | 0.46 | 0.96 | 0.61 |
| Hubei | 10.75 | 11.42 | 17.49 | 11.48 | 5.20 | 5.56 | 8.54 | 5.61 | 0.97 | 1.01 | 1.54 | 1.01 |
| Hunan | 14.28 | 16.09 | 16.35 | 15.24 | 6.91 | 7.84 | 7.98 | 7.44 | 1.29 | 1.43 | 1.44 | 1.34 |
| Jilin | 5.15 | 4.54 | 13.60 | 4.21 | 2.49 | 2.21 | 6.64 | 2.06 | 0.46 | 0.40 | 1.19 | 0.37 |
| Jiangsu | 29.39 | 27.72 | 40.92 | 30.82 | 23.65 | 22.33 | 33.32 | 25.09 | 2.87 | 2.61 | 3.64 | 2.74 |
| Jiangxi | 7.60 | 8.59 | 9.46 | 9.33 | 3.68 | 4.19 | 4.62 | 4.56 | 0.68 | 0.76 | 0.83 | 0.82 |
| Liaoning | 10.94 | 10.23 | 25.03 | 11.31 | 8.23 | 7.70 | 19.06 | 8.62 | 1.63 | 1.47 | 3.40 | 1.54 |
| Inner Mongolia | 6.83 | 7.87 | 10.02 | 8.74 | 1.34 | 1.56 | 2.02 | 1.76 | 1.48 | 1.71 | 2.12 | 1.85 |
| Ningxia | 1.32 | 1.60 | 1.81 | 2.11 | 0.26 | 0.32 | 0.36 | 0.43 | 0.29 | 0.35 | 0.38 | 0.45 |
| Qinghai | 1.81 | 1.70 | 2.04 | 2.32 | 0.35 | 0.34 | 0.41 | 0.47 | 0.39 | 0.37 | 0.43 | 0.49 |
| Shandong | 20.99 | 20.86 | 35.01 | 26.57 | 15.80 | 15.71 | 26.66 | 20.23 | 3.13 | 3.00 | 4.76 | 3.61 |
| Shanxi | 6.71 | 6.60 | 10.70 | 7.88 | 3.25 | 3.22 | 5.23 | 3.85 | 0.60 | 0.59 | 0.94 | 0.69 |
| Shaanxi | 10.82 | 12.11 | 12.59 | 12.97 | 2.12 | 2.40 | 2.54 | 2.61 | 2.34 | 2.63 | 2.66 | 2.74 |
| Shanghai | 9.21 | 9.04 | 6.07 | 5.72 | 4.62 | 4.54 | 3.08 | 2.91 | 2.82 | 2.68 | 1.70 | 1.60 |
| Sichuan | 8.03 | 8.49 | 10.84 | 9.86 | 3.09 | 3.31 | 4.29 | 3.91 | 5.12 | 5.44 | 6.75 | 6.14 |
| Tianjin | 2.39 | 2.62 | 2.92 | 2.83 | 1.80 | 1.97 | 2.23 | 2.16 | 0.36 | 0.38 | 0.40 | 0.39 |
| Tibet | 0.16 | 0.11 | 0.14 | 0.13 | 0.06 | 0.04 | 0.06 | 0.05 | 0.10 | 0.07 | 0.09 | 0.08 |
| Xinjiang | 9.09 | 7.81 | 17.09 | 10.81 | 1.78 | 1.55 | 3.45 | 2.18 | 1.97 | 1.70 | 3.61 | 2.28 |
| Yunnan | 5.16 | 5.29 | 5.47 | 5.33 | 1.98 | 2.06 | 2.17 | 2.11 | 3.29 | 3.39 | 3.40 | 3.32 |
| Zhejiang | 10.21 | 9.78 | 10.86 | 10.29 | 7.68 | 7.37 | 8.27 | 7.83 | 1.52 | 1.41 | 1.48 | 1.40 |
| Chongqing | 2.45 | 2.10 | 2.90 | 3.11 | 0.94 | 0.82 | 1.15 | 1.23 | 1.56 | 1.35 | 1.81 | 1.94 |

# Table.S3 Provincial level O_3_ attributable economic loss during 2015 – 2018 (billion Yuan)

| Province | All-cause mortality | | | | Card-mortality | | | | Respir-mortality | | | |
| --- | --- | --- | --- | --- | --- | --- | --- | --- | --- | --- | --- | --- |
|  | 2015 | 2016 | 2017 | 2018 | 2015 | 2016 | 2017 | 2018 | 2015 | 2016 | 2017 | 2018 |
| Anhui | 9.29 | 11.89 | 15.08 | 17.58 | 4.50 | 5.79 | 7.36 | 8.58 | 0.84 | 1.05 | 1.32 | 1.54 |
| Beijing | 8.95 | 9.95 | 11.38 | 13.41 | 6.73 | 7.49 | 8.67 | 10.21 | 1.33 | 1.43 | 1.55 | 1.82 |
| Fujian | 7.90 | 10.34 | 15.15 | 15.71 | 5.95 | 7.79 | 11.54 | 11.97 | 1.18 | 1.49 | 2.06 | 2.14 |
| Gansu | 5.97 | 6.47 | 7.61 | 8.54 | 1.17 | 1.28 | 1.53 | 1.72 | 1.29 | 1.41 | 1.61 | 1.80 |
| Guangdong | 44.70 | 44.81 | 60.63 | 57.72 | 30.91 | 31.01 | 42.42 | 40.39 | 6.54 | 6.33 | 8.09 | 7.70 |
| Guangxi | 3.89 | 3.91 | 6.02 | 4.14 | 1.50 | 1.53 | 2.39 | 1.64 | 2.48 | 2.50 | 3.75 | 2.58 |
| Guizhou | 2.68 | 3.11 | 3.57 | 3.78 | 1.03 | 1.21 | 1.42 | 1.50 | 1.71 | 1.99 | 2.23 | 2.36 |
| Hainan | 0.58 | 0.63 | 0.72 | 0.70 | 0.44 | 0.47 | 0.55 | 0.54 | 0.09 | 0.09 | 0.10 | 0.10 |
| Hebei | 14.14 | 17.75 | 21.68 | 20.93 | 10.64 | 13.37 | 16.51 | 15.94 | 2.11 | 2.56 | 2.95 | 2.85 |
| Henan | 20.55 | 26.39 | 30.81 | 35.04 | 9.95 | 12.86 | 15.05 | 17.11 | 1.85 | 2.34 | 2.71 | 3.08 |
| Heilongjiang | 7.07 | 5.78 | 13.60 | 8.81 | 3.42 | 2.82 | 6.64 | 4.30 | 0.64 | 0.51 | 1.19 | 0.77 |
| Hubei | 14.80 | 16.04 | 28.99 | 19.24 | 7.16 | 7.81 | 14.16 | 9.40 | 1.33 | 1.42 | 2.55 | 1.69 |
| Hunan | 15.29 | 18.32 | 19.47 | 19.07 | 7.40 | 8.92 | 9.51 | 9.31 | 1.38 | 1.62 | 1.71 | 1.67 |
| Jilin | 7.06 | 6.58 | 18.29 | 5.83 | 3.42 | 3.20 | 8.93 | 2.85 | 0.64 | 0.58 | 1.61 | 0.51 |
| Jiangsu | 56.85 | 57.36 | 91.93 | 73.45 | 45.75 | 46.20 | 74.85 | 59.80 | 5.54 | 5.41 | 8.18 | 6.54 |
| Jiangxi | 7.52 | 9.25 | 10.89 | 11.42 | 3.64 | 4.51 | 5.32 | 5.58 | 0.68 | 0.82 | 0.96 | 1.00 |
| Liaoning | 17.97 | 14.03 | 32.04 | 17.82 | 13.53 | 10.57 | 24.40 | 13.57 | 2.68 | 2.02 | 4.36 | 2.42 |
| Inner Mongolia | 12.17 | 14.41 | 16.37 | 14.25 | 2.38 | 2.86 | 3.30 | 2.87 | 2.63 | 3.13 | 3.46 | 3.01 |
| Ningxia | 1.53 | 2.00 | 2.36 | 2.91 | 0.30 | 0.40 | 0.48 | 0.59 | 0.33 | 0.43 | 0.50 | 0.62 |
| Qinghai | 2.01 | 2.01 | 2.49 | 2.69 | 0.39 | 0.40 | 0.50 | 0.54 | 0.44 | 0.44 | 0.52 | 0.57 |
| Shandong | 34.14 | 35.34 | 62.30 | 49.81 | 25.70 | 26.62 | 47.45 | 37.93 | 5.09 | 5.09 | 8.47 | 6.77 |
| Shanxi | 6.54 | 6.79 | 11.17 | 9.79 | 3.17 | 3.31 | 5.46 | 4.78 | 0.59 | 0.60 | 0.98 | 0.86 |
| Shaanxi | 12.89 | 15.16 | 17.56 | 19.68 | 2.52 | 3.01 | 3.54 | 3.97 | 2.79 | 3.29 | 3.71 | 4.16 |
| Shanghai | 21.75 | 23.62 | 16.78 | 16.87 | 10.91 | 11.86 | 8.52 | 8.56 | 6.66 | 6.99 | 4.69 | 4.72 |
| Sichuan | 8.67 | 10.06 | 14.86 | 13.09 | 3.33 | 3.92 | 5.89 | 5.19 | 5.53 | 6.44 | 9.25 | 8.15 |
| Tianjin | 5.76 | 6.67 | 7.73 | 7.63 | 4.33 | 5.03 | 5.89 | 5.81 | 0.86 | 0.96 | 1.05 | 1.04 |
| Tibet | 0.21 | 0.15 | 0.20 | 0.20 | 0.08 | 0.06 | 0.08 | 0.08 | 0.13 | 0.10 | 0.13 | 0.13 |
| Xinjiang | 8.80 | 8.12 | 21.92 | 13.72 | 1.72 | 1.61 | 4.42 | 2.77 | 1.90 | 1.76 | 4.63 | 2.90 |
| Yunnan | 4.09 | 4.52 | 4.99 | 5.16 | 1.57 | 1.76 | 1.98 | 2.04 | 2.61 | 2.90 | 3.10 | 3.21 |
| Zhejiang | 20.75 | 19.44 | 23.03 | 23.29 | 15.62 | 14.64 | 17.54 | 17.73 | 3.09 | 2.80 | 3.13 | 3.17 |
| Chongqing | 3.24 | 3.02 | 4.48 | 4.98 | 1.25 | 1.18 | 1.77 | 1.97 | 2.07 | 1.94 | 2.79 | 3.10 |

# Table.S4 Provincial level O_3_ attributable GDP impact during 2015 – 2018 (%)

| Province | All-cause mortality | | | | Card-mortality | | | | Respir-mortality | | | |
| --- | --- | --- | --- | --- | --- | --- | --- | --- | --- | --- | --- | --- |
|  | 2015 | 2016 | 2017 | 2018 | 2015 | 2016 | 2017 | 2018 | 2015 | 2016 | 2017 | 2018 |
| Anhui | 0.41 | 0.48 | 0.55 | 0.58 | 0.20 | 0.23 | 0.27 | 0.28 | 0.04 | 0.04 | 0.05 | 0.05 |
| Beijing | 0.39 | 0.39 | 0.41 | 0.44 | 0.29 | 0.29 | 0.31 | 0.34 | 0.06 | 0.06 | 0.06 | 0.06 |
| Fujian | 0.30 | 0.36 | 0.47 | 0.44 | 0.23 | 0.27 | 0.36 | 0.33 | 0.05 | 0.05 | 0.06 | 0.06 |
| Gansu | 0.88 | 0.91 | 1.04 | 1.04 | 0.17 | 0.18 | 0.21 | 0.21 | 0.19 | 0.20 | 0.22 | 0.22 |
| Guangdong | 0.56 | 0.52 | 0.63 | 0.57 | 0.39 | 0.36 | 0.44 | 0.40 | 0.08 | 0.07 | 0.08 | 0.08 |
| Guangxi | 0.23 | 0.21 | 0.29 | 0.20 | 0.09 | 0.08 | 0.12 | 0.08 | 0.15 | 0.14 | 0.18 | 0.13 |
| Guizhou | 0.23 | 0.24 | 0.24 | 0.24 | 0.09 | 0.09 | 0.10 | 0.10 | 0.15 | 0.15 | 0.15 | 0.15 |
| Hainan | 0.38 | 0.39 | 0.40 | 0.35 | 0.29 | 0.29 | 0.31 | 0.27 | 0.06 | 0.06 | 0.05 | 0.05 |
| Hebei | 0.46 | 0.54 | 0.63 | 0.61 | 0.35 | 0.40 | 0.48 | 0.46 | 0.07 | 0.08 | 0.09 | 0.08 |
| Henan | 0.56 | 0.65 | 0.69 | 0.74 | 0.27 | 0.32 | 0.34 | 0.36 | 0.05 | 0.06 | 0.06 | 0.06 |
| Heilongjiang | 0.46 | 0.37 | 0.88 | 0.50 | 0.22 | 0.18 | 0.43 | 0.25 | 0.04 | 0.03 | 0.08 | 0.04 |
| Hubei | 0.50 | 0.50 | 0.81 | 0.50 | 0.24 | 0.24 | 0.40 | 0.24 | 0.05 | 0.04 | 0.07 | 0.04 |
| Hunan | 0.50 | 0.55 | 0.55 | 0.51 | 0.24 | 0.27 | 0.27 | 0.25 | 0.05 | 0.05 | 0.05 | 0.04 |
| Jilin | 0.48 | 0.43 | 1.25 | 0.39 | 0.23 | 0.21 | 0.61 | 0.19 | 0.04 | 0.04 | 0.11 | 0.03 |
| Jiangsu | 0.79 | 0.73 | 1.04 | 0.78 | 0.64 | 0.59 | 0.85 | 0.63 | 0.08 | 0.07 | 0.09 | 0.07 |
| Jiangxi | 0.45 | 0.50 | 0.53 | 0.52 | 0.22 | 0.24 | 0.26 | 0.25 | 0.04 | 0.04 | 0.05 | 0.05 |
| Liaoning | 0.63 | 0.62 | 1.35 | 0.69 | 0.47 | 0.47 | 1.03 | 0.52 | 0.09 | 0.09 | 0.18 | 0.09 |
| Inner Mongolia | 0.59 | 0.67 | 0.96 | 0.82 | 0.12 | 0.13 | 0.19 | 0.17 | 0.13 | 0.15 | 0.20 | 0.17 |
| Ningxia | 0.52 | 0.63 | 0.67 | 0.77 | 0.10 | 0.13 | 0.14 | 0.16 | 0.11 | 0.14 | 0.14 | 0.16 |
| Qinghai | 0.84 | 0.75 | 0.93 | 0.96 | 0.16 | 0.15 | 0.19 | 0.19 | 0.18 | 0.16 | 0.20 | 0.20 |
| Shandong | 0.54 | 0.52 | 0.85 | 0.64 | 0.41 | 0.39 | 0.65 | 0.49 | 0.08 | 0.08 | 0.12 | 0.09 |
| Shanxi | 0.52 | 0.52 | 0.75 | 0.58 | 0.25 | 0.25 | 0.36 | 0.28 | 0.05 | 0.05 | 0.07 | 0.05 |
| Shaanxi | 0.72 | 0.80 | 0.81 | 0.82 | 0.14 | 0.16 | 0.16 | 0.16 | 0.16 | 0.17 | 0.17 | 0.17 |
| Shanghai | 0.87 | 0.84 | 0.56 | 0.52 | 0.44 | 0.42 | 0.28 | 0.26 | 0.27 | 0.25 | 0.16 | 0.14 |
| Sichuan | 0.27 | 0.29 | 0.38 | 0.31 | 0.10 | 0.11 | 0.15 | 0.12 | 0.17 | 0.18 | 0.24 | 0.19 |
| Tianjin | 0.34 | 0.37 | 0.42 | 0.41 | 0.26 | 0.28 | 0.32 | 0.31 | 0.05 | 0.05 | 0.06 | 0.06 |
| Tibet | 0.41 | 0.27 | 0.31 | 0.29 | 0.16 | 0.10 | 0.12 | 0.11 | 0.26 | 0.17 | 0.19 | 0.18 |
| Xinjiang | 0.85 | 0.83 | 2.04 | 1.15 | 0.17 | 0.17 | 0.41 | 0.23 | 0.18 | 0.18 | 0.43 | 0.24 |
| Yunnan | 0.29 | 0.30 | 0.30 | 0.29 | 0.11 | 0.12 | 0.12 | 0.11 | 0.19 | 0.19 | 0.19 | 0.18 |
| Zhejiang | 0.41 | 0.41 | 0.44 | 0.41 | 0.31 | 0.31 | 0.34 | 0.31 | 0.06 | 0.06 | 0.06 | 0.06 |
| Chongqing | 0.21 | 0.17 | 0.23 | 0.24 | 0.08 | 0.07 | 0.09 | 0.10 | 0.13 | 0.11 | 0.14 | 0.15 |

# Table.S5 Differences of health impacts with and without exposure factors at provincial and national level (person) / (%)

| Provinces | 2015 All-cause | 2016 All-cause | 2017 All-cause | 2018 All-cause |
| --- | --- | --- | --- | --- |
| Anhui | -220 (-2.27) | -287 (-2.47) | -334 (-2.43) | -368 (-2.51) |
| Beijing | 33 (0.91) | 35 (0.92) | 37 (0.92) | 40 (0.92) |
| Fujian | -153 (-3.03) | -189 (-3.06) | -254 (-3.06) | -253 (-3.19) |
| Gansu | 616 (7.79) | 643 (7.8) | 732 (7.79) | 755 (7.78) |
| Guangdong | -1272 (-4.34) | -1242 (-4.37) | -1500 (-4.17) | -1458 (-4.41) |
| Guangxi | 77 (1.94) | 82 (2.13) | 111 (2.21) | 71 (1.90) |
| Guizhou | 4 (0.13) | 4 (0.12) | 4 (0.12) | 5 (0.14) |
| Hainan | 30 (7.61) | 32 (8.08) | 34 (8.11) | 30 (7.89) |
| Hebei | -105 (-0.82) | -130 (-0.86) | -143 (-0.8) | -137 (-0.81) |
| Henan | 1160 (6.07) | 1385 (6.07) | 1506 (6.10) | 1615 (6.04) |
| Heilongjiang | -20 (-0.30) | -26 (-0.49) | -45 (-0.41) | -29 (-0.42) |
| Hubei | 495 (4.83) | 541 (4.97) | 827 (4.96) | 526 (4.80) |
| Hunan | 52 (0.37) | 65 (0.41) | 70 (0.43) | 55 (0.36) |
| Jilin | -190 (-3.55) | -166 (-3.52) | -519 (-3.67) | -160 (-3.65) |
| Jiangsu | -1673 (-5.39) | -1564 (-5.34) | -2315 (-5.35) | -1753 (-5.38) |
| Jiangxi | -152 (-1.97) | -164 (-1.87) | -170 (-1.77) | -199 (-2.09) |
| Liaoning | 131 (1.22) | 101 (1.00) | 250 (1.01) | 109 (0.97) |
| Inner Mongolia | 341 (5.25) | 386 (5.16) | 490 (5.14) | 429 (5.16) |
| Ningxia | -28 (-2.07) | -34 (-2.09) | -39 (-2.10) | -46 (-2.11) |
| Qinghai | -43 (-2.31) | -39 (-2.21) | -47 (-2.27) | -54 (-2.26) |
| Shandong | -351 (-1.64) | -364 (-1.72) | -607 (-1.70) | -457 (-1.69) |
| Shanxi | 188 (2.88) | 179 (2.78) | 302 (2.90) | 224 (2.92) |
| Shaanxi | 56 (0.52) | 62 (0.52) | 64 (0.51) | 68 (0.53) |
| Shanghai | -441 (-4.57) | -430 (-4.54) | -290 (-4.55) | -273 (-4.55) |
| Sichuan | 65 (0.81) | 76 (0.90) | 96 (0.89) | 98 (1.00) |
| Tianjin | 13 (0.55) | 12 (0.47) | 14 (0.48) | 15 (0.53) |
| Tibet | 11 (7.37) | 8 (7.72) | 10 (7.71) | 10 (7.79) |
| Xinjiang | 771 (9.27) | 663 (9.28) | 1453 (9.29) | 919 (9.28) |
| Yunnan | 387 (8.12) | 401 (8.21) | 421 (8.34) | 402 (8.16) |
| Zhejiang | -259 (-2.47) | -240 (-2.40) | -266 (-2.39) | -259 (-2.46) |
| Chongqing | -190 (-7.19) | -163 (-7.17) | -223 (-7.15) | -240 (-7.15) |
| National | -667 (-0.24) | -361 (-0.13) | -331 (-0.09) | -316 (-0.10) |

Note**:** the sign ‘-’ represent overestimation

# Table.S6 Monthly proportion of the deaths in Chinese province (%)

| Province | January | February | March | April | May | June | July | August | September | October | November | December |
| --- | --- | --- | --- | --- | --- | --- | --- | --- | --- | --- | --- | --- |
| Anhui | 7.3 | 8.6 | 9.1 | 8.6 | 8.0 | 7.7 | 8.5 | 8.3 | 8.2 | 9.5 | 8.1 | 8.2 |
| Beijing | 8.6 | 7.6 | 8.2 | 8.1 | 8.1 | 7.0 | 8.5 | 8.4 | 8.5 | 9.6 | 8.8 | 8.8 |
| Fujian | 8.5 | 8.3 | 9.3 | 8.4 | 8.2 | 7.9 | 8.2 | 8.3 | 7.9 | 9.0 | 7.8 | 8.3 |
| Gansu | 6.9 | 8.5 | 10.1 | 8.8 | 8.6 | 7.8 | 8.2 | 9.2 | 7.4 | 8.1 | 8.0 | 8.6 |
| Guangdong | 8.4 | 8.5 | 9.4 | 8.5 | 8.5 | 7.9 | 8.2 | 8.0 | 7.6 | 8.1 | 8.5 | 8.5 |
| Guangxi | 6.4 | 7.8 | 8.8 | 8.3 | 8.7 | 8.2 | 8.4 | 8.8 | 8.3 | 9.8 | 7.9 | 8.8 |
| Guizhou | 7.4 | 8.2 | 8.7 | 8.3 | 8.6 | 8.4 | 8.1 | 8.3 | 7.7 | 9.7 | 7.9 | 8.7 |
| Hainan | 6.2 | 7.1 | 8.3 | 7.9 | 8.4 | 8.9 | 8.5 | 9.1 | 9.3 | 10.6 | 7.6 | 8.2 |
| Hebei | 9.1 | 9.2 | 9.3 | 7.9 | 7.8 | 7.2 | 8.0 | 7.5 | 7.4 | 8.3 | 9.0 | 9.5 |
| Henan | 7.4 | 9.3 | 10.0 | 8.7 | 8.2 | 7.8 | 8.7 | 8.4 | 8.1 | 8.6 | 6.9 | 8.0 |
| Heilongjiang | 7.1 | 8.0 | 9.4 | 9.6 | 8.9 | 9.0 | 9.1 | 8.4 | 8.2 | 8.2 | 6.8 | 7.5 |
| Hubei | 7.6 | 8.4 | 8.7 | 8.2 | 8.4 | 7.5 | 8.3 | 8.8 | 8.5 | 9.4 | 7.6 | 8.5 |
| Hunan | 7.6 | 8.5 | 8.5 | 8.8 | 8.8 | 7.9 | 8.2 | 8.8 | 8.1 | 9.1 | 7.2 | 8.4 |
| Jilin | 7.4 | 8.4 | 9.5 | 9.9 | 9.2 | 8.7 | 8.9 | 8.6 | 8.3 | 8.1 | 6.0 | 7.1 |
| Jiangsu | 9.0 | 8.7 | 9.0 | 8.4 | 7.9 | 7.2 | 7.6 | 8.3 | 7.6 | 8.8 | 8.5 | 9.1 |
| Jiangxi | 6.6 | 8.5 | 8.6 | 8.3 | 8.6 | 7.9 | 8.2 | 8.6 | 7.8 | 9.1 | 8.2 | 9.7 |
| Liaoning | 8.7 | 8.0 | 9.0 | 8.8 | 8.5 | 7.7 | 7.8 | 8.1 | 8.4 | 8.8 | 8.0 | 8.3 |
| Inner Mongolia | 6.7 | 7.7 | 9.2 | 8.9 | 8.9 | 8.3 | 8.7 | 8.3 | 8.6 | 10.0 | 7.0 | 7.6 |
| Ningxia | 7.3 | 7.8 | 8.9 | 8.3 | 8.8 | 8.3 | 7.8 | 8.6 | 8.1 | 9.7 | 8.4 | 8.0 |
| Qinghai | 5.9 | 6.9 | 8.2 | 21.0 | 8.0 | 7.4 | 7.8 | 7.3 | 6.9 | 7.7 | 6.8 | 6.2 |
| Shandong | 9.6 | 9.6 | 9.4 | 8.2 | 7.9 | 7.6 | 8.2 | 7.8 | 7.9 | 7.8 | 7.7 | 8.5 |
| Shanxi | 7.7 | 8.9 | 9.8 | 8.6 | 8.3 | 7.6 | 8.3 | 7.8 | 7.5 | 8.1 | 8.3 | 9.3 |
| Shaanxi | 7.5 | 9.0 | 9.4 | 8.6 | 8.5 | 7.5 | 8.5 | 8.5 | 7.8 | 8.6 | 7.6 | 8.6 |
| Shanghai | 10.0 | 8.5 | 9.1 | 8.2 | 7.8 | 7.1 | 7.2 | 7.9 | 7.7 | 8.9 | 8.1 | 9.5 |
| Sichuan | 8.2 | 8.2 | 9.0 | 8.2 | 8.3 | 7.9 | 8.4 | 8.8 | 8.1 | 8.4 | 7.6 | 8.9 |
| Tianjin | 8.8 | 7.6 | 8.4 | 8.6 | 8.2 | 7.5 | 8.4 | 8.3 | 8.6 | 9.0 | 8.3 | 8.3 |
| Xizang | 6.1 | 8.2 | 10.6 | 10.5 | 9.3 | 9.0 | 11.8 | 10.1 | 7.7 | 5.4 | 5.4 | 6.1 |
| Xinjiang | 6.2 | 7.4 | 8.9 | 9.0 | 9.1 | 8.4 | 9.1 | 9.2 | 8.6 | 10.1 | 7.1 | 6.8 |
| Yunnan | 7.0 | 7.9 | 8.7 | 8.7 | 8.4 | 8.4 | 8.2 | 8.7 | 8.2 | 9.8 | 7.4 | 8.7 |
| Zhejiang | 9.7 | 8.7 | 9.0 | 8.1 | 8.0 | 7.1 | 7.4 | 7.9 | 7.7 | 9.2 | 8.3 | 9.1 |
| Chongqing | 7.9 | 8.1 | 8.7 | 8.2 | 8.5 | 8.0 | 9.0 | 9.2 | 8.0 | 8.9 | 7.2 | 8.4 |
